# Supplementary material for: Template-Based Assembly of Proteomic Short Reads For De Novo Antibody Sequencing and Repertoire Profiling
Source: Anal Chem. 2022 Jul 14;94(29):10391–9. doi: 10.1021/acs.analchem.2c01300 (PMC9330293; doi:10.1021/acs.analchem.2c01300)
Supplement: Supplementary file 1 — ac2c01300_si_001.pdf [file ac2c01300_si_001.pdf]

Supplementary Information to:

Template-based assembly of proteomic short reads for *de novo* antibody sequencing and repertoire profiling

Authors:

Douwe Schulte[1], Weiwei Peng[1], Joost Snijder[1]*

* corresponding author: j.snijder@uu.nl

Affiliations:

[1] Biomolecular Mass Spectrometry and Proteomics, Bijvoet Center for Biomolecular Research and Utrecht Institute of Pharmaceutical Sciences, Utrecht University, Padualaan 8, 3584 CH Utrecht, The Netherlands

Contents

Legend of Supplementary Data with Stitch HTML reports

CDRH3 reconstructions of Herceptin and anti-FLAG-M2

Details of sequence variations in F59 Fab

Description of multiple myeloma light chain sequences.

Supplementary Data.

All reported Stitch analyses are provided with the complete output reports. The supplementary data can be unzipped to browse the interactive HTML reports. The corresponding analysis parameters are provided under the ‘Batch File’ menu in each report.

```
A) Herceptin

template   ...DTAVYYCAR                 YFDYWGQGTLVTVSS...
de novo       DTAVYYCSRWGG     SRWGGDGFYAMDYWGQGTLVTVSS

overlap       DTAVYYCSRWGG
                     SRWGGDGFYAMDYWGQGTLVTVSS

ref        ...DTAVYYCSRWGGDGFYAMDYWGQGTLVTVSS...


B) anti-FLAG-M2

template   ...DSAVYYCAR                     YFDYWGQGTTLTVSS...
de novo       DSAVYYCAREKFYGY     VYYCAREKFYGYDYWGQGATLTVSS

overlap       DSAVYYCAREKFYGY
                 VYYCAREKFYGYDYWGQGATLTVSS

ref        ...DSAVYYCAREKFYGYDYWGQGATLTVSS...
```

Supplementary Figure S1. Detailed view of CDRH3 reconstruction by Stitch of the antibodies shown in main text figure 2. Shown are the selected template sequences, aligned reads, found overlap and known reference sequence.

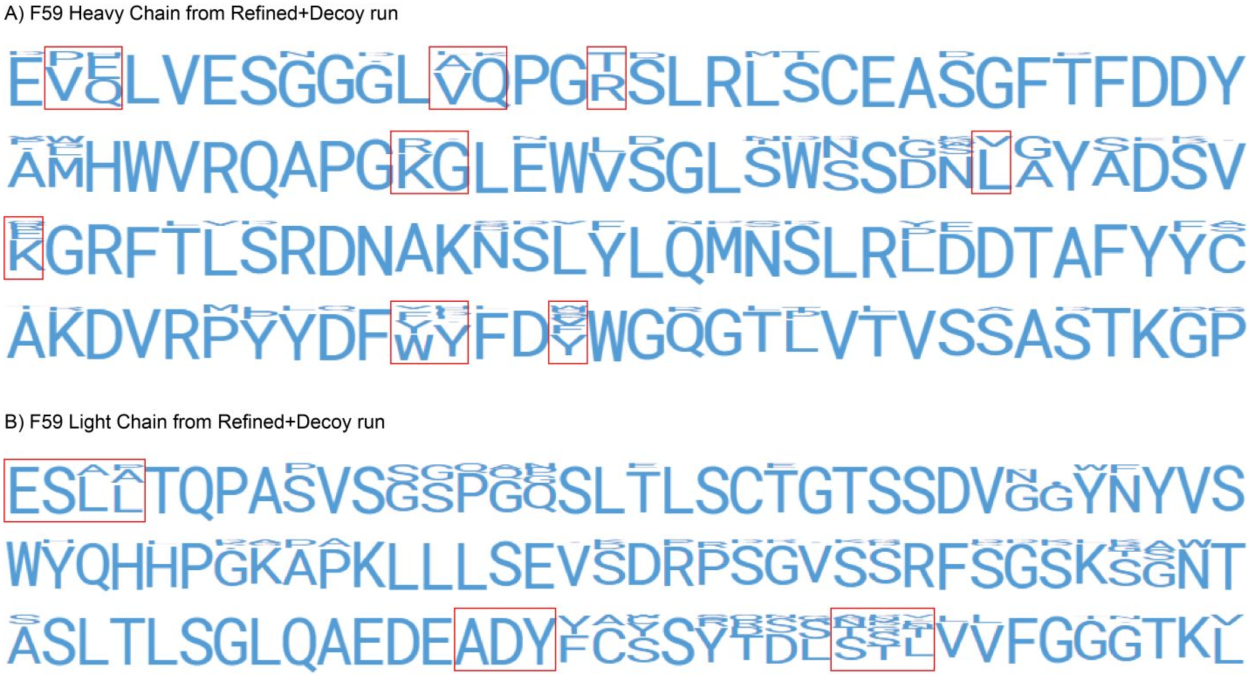

Supplementary Figure S2. Sequence logos of heavy (A) and light (B) chains of F59 Fab from fractionated serum sample from Refined+Decoy Stitch runs. Sequencing error are highlighted in red boxes.

A)

```
>Patient 1 (94.5% Δ -18.3 Da)
de novo   | ESALTQPRSVSGSPGQSVTISCTGTSSDVGGYNYVSWYQQHPGKAPKLMIYDVTKRPSGVPDRFSGSKSGTTASLTISGLQAEDEADYYCCSYAGLDLFVLFGGGTKLTV
reference | GPDLTQPRSVSGSPGQSVTLSCTGTSSDVGGYNYVSWYQQHPGKAPKLMIYDVTKRPSGVPDRFSGSKSGTTASLTISGLQAEDEADYYCCSYAGIDIFVLFGGGTKLTV
            ***                *                                                                           * *

>Patient 5 (A: 99.0% Δ -30.4 Da) & (B; 87.9%)
de novo     | EIVLTQSPGTLSLSPGERATLSCRASQSVSSSYLAWYQQKPGQAPRLLIYDASTRATGIPDRFSGSGSGTDFLLTISSLEPEDFAMYYCQQYGRSPYTFGPGTKVDI
reference A | EIVLTQSPGTLSLSPGERATLSCRASQSVSSSYLAWYQQKPGQAPRLLIYDASTRATGIPDRFSGSGSGADFLLTISSLEPEDFAMYYCQQYGRSPYTFGPGTKVDI
reference B | EIVLSQSPDTLSLSPGERATLSCRADKSVSSNYVAWYQQKPGQAPRLLIYDAFTRATGIPDRFSGSGSETDYTLTISTLEPEDFAVYYCQQYGRSPYTFGPGTKVDI
                  *   *                **    * *                  *               ** **    *       *

>Patient 6 (100.0% Δ -0.5 Da)
de novo   | DIQMTQSPSSLSASVGDRVSITCRASESISSYVNWYQQKPGKAPKLLIYTASSLQSGVPPRFSGSASGTDFTLTISSLQPEDFATYYCQQSYSTPITFGQGTRLEI
reference | DIQMTQSPSSLSASVGDRVSITCRASESISSYVNWYQQKPGKAPKLLIYTASSLQSGVPPRFSGSASGTDFTLTISSLQPEDFATYYCQQSYSTPITFGQGTRLEI

>Patient 7 (94.3% Δ 60.8 Da)
de novo   | DIQMTQSPSSLSASVGDRVTITCQASQDIAKYLNWYQQKPGKPPKLLIYDTSNLETGVPSRFSGSGSGTDFTFTINSLQPEDIATYYCQQYDDFPLTFGPGTKVDI
reference | DIQMTQSPSSLSASVGDRVTITCQASQDLAKYLNWYQQKPGKPPKLLIYDTSNLETGVPSRFSNG-GGTDFTFTINSLQPEDLATYYCQQYDDFPLTFGPGTKVDI
                                        *                                  ****               *

>Patient 8 (99.1% Δ -0.5 Da)
de novo   | DIQMTQSPSTLSASVGDRVTITCRASQSISSSLAWYQQKPGKAPKLLIYDASSLETGVPSRFSGSGSGTEFTLSISSLQPDDFATYYCQHYNSYSLTFGQGTKVEI
reference | DIQMTQSPSTLSASVGDRVTITCRASQSLSSSLAWYQQKPGKAPKLLIYDASSLETGVPSRFSGSGSGTEFTLSISSLQPDDFATYYCQHYNSYSLTFGQGTKVEI
                                        *

>Patient 13 (92.8% Δ -12.5 Da)
de novo   | EPALTQPPSVSGAPGQRVTISCTGSSSNIGAGWDVHWYQQLPGTVPKLLIYADRNRPSGVPERFSGSKSGTDAALAIAGLQAEDEADYYCQSYDSALSGFYVFGTGTKVLV
reference | EAPLTQPPSVSGAPGQRVTLSCTGSSSNLGAGWDVHWYQQLPGTVPKLLIYADRNRPSGVPERFSGSKSGTSATVAIAGLQAEDEADYYCQSYDSALSGFYVFGTGTKVIV
            ***                *        *                                          *  *                                  *

>Patient 15 (99.1% Δ -0.8 Da)
de novo   | DIQMTQSPSTLSASVGDAVTITCRASQSINVWLAWYQQKPGKPPKLLIYEASNLESGVPSRFSGSGSGTEFTLTISSLQPDDFATYYCQQYNSYPYTFGQGAKLEI
reference | DIQMTQSPSTLSASVGDAVTITCRASQSLNVWLAWYQQKPGKPPKLLIYEASNLESGVPSRFSGSGSGTEFTLTISSLQPDDFATYYCQQYNSYPYTFGQGAKLEI
                                        *

>Patient 18 (99.1% Δ -0.7 Da)
de novo   | DIQMTQSPSSLSASVGDRVTITCQASRDISNYLNWYQQKPGKAPMLLIYAASNLQTGVPSRFSGSGSGTDFTFTISSLQPEDIATYYCQQYGNLPLTFGGGTKVEI
reference | DIQMTQSPSSLSASVGDRVTITCQASRDISNYLNWYQQKPGKAPMLLIYAASNLQTGVPSRFSGSGSGTDFTFTISSLQPEDLATYYCQQYGNLPLTFGGGTKVEI
                                                                                              *

>Patient 19 (99.1% Δ -0.8 Da)
de novo   | DIQMTQSPSSLSASVGDRVTITCQASQDIGNYLNWYQQKPGKAPRLLIYDASDLEEGVPSRFSGSGSGTDFTFTISSLQPEDFATYYCQQYHTLPPLTFGGGTKVDV
reference | DIQMTQSPSSLSASVGDRVTITCQASQDLGNYLNWYQQKPGKAPRLLIYDASDLEEGVPSRFSGSGSGTDFTFTISSLQPEDFATYYCQQYHTLPPLTFGGGTKVDV
                                        *

>Patient 20 (99.1% Δ -1.8 Da)
de novo   | DIQMTQSPSTLSTSVGDRVTITCRASQSIRTWLAWYQQKPGKAPKLLIYKASTLETGVPSRFSGSGSGTMFTLTISSLQPEDFATYYCQQYNDYSGTFGQGTKLEI
reference | DIQMTQSPSTLSTSVGDRVTITCRASQSIRTWLAWYQQKPGKAPKLLIYKASTLETGVPSRFSGSGSGTEFTLTISSLQPEDFATYYCQQYNDYSGTFGQGTKLEI
                                                                                 *
```

B)

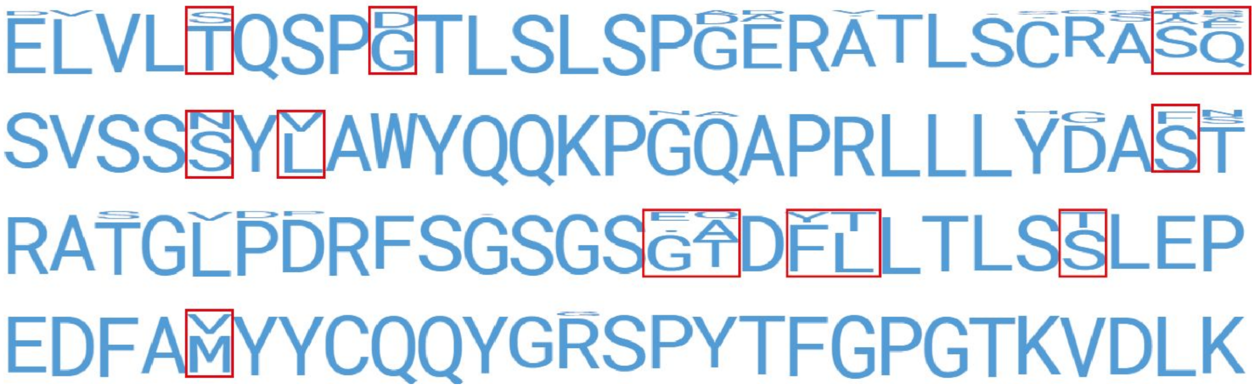

Supplementary Figure S3. A) Stitch reconstruction of light chains from multiple myeloma patient urine (Chamot-Rooke and colleagues, ref 43). CDRs are annotated, sequence conflicts

highlighted by an asterisk (*) and sequence identity in parentheses, along with the mass error of the predicted Stitch sequence compared to the experimentally determined mass of the intact light chain. B) Sequence logo of P5 light chain from Stitch analysis, with highlighted variants in red boxes.
