# Supplementary material for: Template-Based Assembly of Proteomic Short Reads For De Novo Antibody Sequencing and Repertoire Profiling
Source: Anal Chem. 2022 Jul 14;94(29):10391–9. doi: 10.1021/acs.analchem.2c01300 (PMC9330293; doi:10.1021/acs.analchem.2c01300)
Supplement: Supplementary file 2 — ac2c01300_si_002.zip [file ac2c01300_si_002.zip › Schulte_2022_ACS-AC_Stitch_SupplementaryData/2022-06-22@17-20-24 anti-FLAG-M2/report-monoclonal/reads/F1_10082.html]

Details F1\_10082

OverviewUndefined

# Read F1:10082

## Sequence

DTDGSYFKYKLNVQKSNWEQNTFTCSVLHEGLH

## Sequence Length

33

## Meta Information from PEAKS

### Scan Identifier

F1:10082

### Original Sequence (length=49)

D

T

D

G

S

Y

F

K

+58.01

Y

K

L

N

V

Q

K

S

N

W

E

Q

N

T

F

T

C

+58.01

S

V

L

H

E

G

L

H

### Posttranslational Modifications

Carboxymethyl (KW X@N-term); Carboxymethyl

### Source File

20191211\_F1\_Ag5\_peng0013\_SA\_Flag\_Asp\_N.raw

### Fraction

1

### Scan Feature

F1:21278

### De Novo Score

91

### Confidence score

91

### Mass Charge Ratio

1001.9655

### Mass

4003.8323

### Charge

4

### Retention Time

55.66

### Predicted Retention Time

-

### Area

14845000

### Parts Per Million

0.1

### Fragmentation Mode

ETHCD
