# Supplementary material for: Template-Based Assembly of Proteomic Short Reads For De Novo Antibody Sequencing and Repertoire Profiling
Source: Anal Chem. 2022 Jul 14;94(29):10391–9. doi: 10.1021/acs.analchem.2c01300 (PMC9330293; doi:10.1021/acs.analchem.2c01300)
Supplement: Supplementary file 2 — ac2c01300_si_002.zip [file ac2c01300_si_002.zip › Schulte_2022_ACS-AC_Stitch_SupplementaryData/2022-06-22@17-20-24 anti-FLAG-M2/report-monoclonal/reads/F1_10123.html]

Details F1\_10123

OverviewUndefined

# Read F1:10123

## Sequence

DETTLTADPSSSTAYMEKQLTSY

## Sequence Length

23

## Meta Information from PEAKS

### Scan Identifier

F1:10123

### Original Sequence (length=31)

D

E

T

T

L

T

A

D

P

S

S

S

T

A

Y

M

E

K

+58.01

Q

L

T

S

Y

### Posttranslational Modifications

Carboxymethyl (KW X@N-term)

### Source File

20191211\_F1\_Ag5\_peng0013\_SA\_Flag\_Asp\_N.raw

### Fraction

1

### Scan Feature

F1:17301

### De Novo Score

94

### Confidence score

94

### Mass Charge Ratio

866.3964

### Mass

2596.1375

### Charge

3

### Retention Time

56.09

### Predicted Retention Time

-

### Area

2986600

### Parts Per Million

11.5

### Fragmentation Mode

ETHCD
