# Supplementary material for: Template-Based Assembly of Proteomic Short Reads For De Novo Antibody Sequencing and Repertoire Profiling
Source: Anal Chem. 2022 Jul 14;94(29):10391–9. doi: 10.1021/acs.analchem.2c01300 (PMC9330293; doi:10.1021/acs.analchem.2c01300)
Supplement: Supplementary file 2 — ac2c01300_si_002.zip [file ac2c01300_si_002.zip › Schulte_2022_ACS-AC_Stitch_SupplementaryData/2022-06-22@17-20-24 anti-FLAG-M2/report-monoclonal/reads/F1_10203.html]

Details F1\_10203

OverviewUndefined

# Read F1:10203

## Sequence

DPSSSTASHSELNSLTSEDSAVYYCAR

## Sequence Length

27

## Meta Information from PEAKS

### Scan Identifier

F1:10203

### Original Sequence (length=35)

D

P

S

S

S

T

A

S

H

S

E

L

N

S

L

T

S

E

D

S

A

V

Y

Y

C

+58.01

A

R

### Posttranslational Modifications

Carboxymethyl

### Source File

20191211\_F1\_Ag5\_peng0013\_SA\_Flag\_Asp\_N.raw

### Fraction

1

### Scan Feature

F1:20964

### De Novo Score

90

### Confidence score

90

### Mass Charge Ratio

979.0913

### Mass

2934.2461

### Charge

3

### Retention Time

56.4

### Predicted Retention Time

-

### Area

1392800

### Parts Per Million

2

### Fragmentation Mode

ETHCD
