# Supplementary material for: Template-Based Assembly of Proteomic Short Reads For De Novo Antibody Sequencing and Repertoire Profiling
Source: Anal Chem. 2022 Jul 14;94(29):10391–9. doi: 10.1021/acs.analchem.2c01300 (PMC9330293; doi:10.1021/acs.analchem.2c01300)
Supplement: Supplementary file 2 — ac2c01300_si_002.zip [file ac2c01300_si_002.zip › Schulte_2022_ACS-AC_Stitch_SupplementaryData/2022-06-22@17-20-24 anti-FLAG-M2/report-monoclonal/reads/F1_10255.html]

Details F1\_10255

OverviewUndefined

# Read F1:10255

## Sequence

DALGVYYCFQGSHVPLPWVGLDAG

## Sequence Length

24

## Meta Information from PEAKS

### Scan Identifier

F1:10255

### Original Sequence (length=48)

D

+58.01

A

L

G

V

Y

Y

C

+58.01

F

Q

G

S

H

V

P

L

P

W

+15.99

V

G

L

D

A

G

### Posttranslational Modifications

Carboxymethyl (KW X@N-term); Carboxymethyl; Oxidation (HW)

### Source File

20191211\_F1\_Ag5\_peng0013\_SA\_Flag\_Asp\_N.raw

### Fraction

1

### Scan Feature

F1:18281

### De Novo Score

91

### Confidence score

91

### Mass Charge Ratio

899.4167

### Mass

2695.2263

### Charge

3

### Retention Time

56.77

### Predicted Retention Time

-

### Area

63076000

### Parts Per Million

0.8

### Fragmentation Mode

ETHCD
