# Supplementary material for: Template-Based Assembly of Proteomic Short Reads For De Novo Antibody Sequencing and Repertoire Profiling
Source: Anal Chem. 2022 Jul 14;94(29):10391–9. doi: 10.1021/acs.analchem.2c01300 (PMC9330293; doi:10.1021/acs.analchem.2c01300)
Supplement: Supplementary file 2 — ac2c01300_si_002.zip [file ac2c01300_si_002.zip › Schulte_2022_ACS-AC_Stitch_SupplementaryData/2022-06-22@17-20-24 anti-FLAG-M2/report-monoclonal/reads/F1_10354.html]

Details F1\_10354

OverviewUndefined

# Read F1:10354

## Sequence

DLGVYYCYQGSHVPYTFGGGTKL

## Sequence Length

23

## Meta Information from PEAKS

### Scan Identifier

F1:10354

### Original Sequence (length=31)

D

L

G

V

Y

Y

C

+58.01

Y

Q

G

S

H

V

P

Y

T

F

G

G

G

T

K

L

### Posttranslational Modifications

Carboxymethyl

### Source File

20191211\_F1\_Ag5\_peng0013\_SA\_Flag\_Asp\_N.raw

### Fraction

1

### Scan Feature

F1:17151

### De Novo Score

97

### Confidence score

97

### Mass Charge Ratio

861.7345

### Mass

2582.1787

### Charge

3

### Retention Time

57.19

### Predicted Retention Time

-

### Area

27353000

### Parts Per Million

1.1

### Fragmentation Mode

ETHCD
