# Supplementary material for: Template-Based Assembly of Proteomic Short Reads For De Novo Antibody Sequencing and Repertoire Profiling
Source: Anal Chem. 2022 Jul 14;94(29):10391–9. doi: 10.1021/acs.analchem.2c01300 (PMC9330293; doi:10.1021/acs.analchem.2c01300)
Supplement: Supplementary file 2 — ac2c01300_si_002.zip [file ac2c01300_si_002.zip › Schulte_2022_ACS-AC_Stitch_SupplementaryData/2022-06-22@17-20-24 anti-FLAG-M2/report-monoclonal/reads/F1_10385.html]

Details F1\_10385

OverviewUndefined

# Read F1:10385

## Sequence

DLTVEWQW

## Sequence Length

8

## Meta Information from PEAKS

### Scan Identifier

F1:10385

### Original Sequence (length=16)

D

L

T

V

E

W

Q

W

+15.99

### Posttranslational Modifications

Oxidation (HW)

### Source File

20191211\_F1\_Ag5\_peng0013\_SA\_Flag\_Asp\_N.raw

### Fraction

1

### Scan Feature

-

### De Novo Score

97

### Confidence score

97

### Mass Charge Ratio

546.7534

### Mass

1091.4924

### Charge

2

### Retention Time

57.55

### Predicted Retention Time

-

### Area

0

### Fragmentation Mode

ETHCD

### Also found in scans

F1:11822
