# Supplementary material for: Template-Based Assembly of Proteomic Short Reads For De Novo Antibody Sequencing and Repertoire Profiling
Source: Anal Chem. 2022 Jul 14;94(29):10391–9. doi: 10.1021/acs.analchem.2c01300 (PMC9330293; doi:10.1021/acs.analchem.2c01300)
Supplement: Supplementary file 2 — ac2c01300_si_002.zip [file ac2c01300_si_002.zip › Schulte_2022_ACS-AC_Stitch_SupplementaryData/2022-06-22@17-20-24 anti-FLAG-M2/report-monoclonal/reads/F1_10426.html]

Details F1\_10426

OverviewUndefined

# Read F1:10426

## Sequence

DLPCMNMCTVPEVSSVFLFPPKPK

## Sequence Length

24

## Meta Information from PEAKS

### Scan Identifier

F1:10426

### Original Sequence (length=48)

D

L

P

C

+58.01

M

+15.99

N

M

C

+58.01

T

V

P

E

V

S

S

V

F

L

F

P

P

K

P

K

### Posttranslational Modifications

Carboxymethyl; Oxidation (M)

### Source File

20191211\_F1\_Ag5\_peng0013\_SA\_Flag\_Asp\_N.raw

### Fraction

1

### Scan Feature

F1:12115

### De Novo Score

93

### Confidence score

93

### Mass Charge Ratio

703.5836

### Mass

2810.304

### Charge

4

### Retention Time

57.82

### Predicted Retention Time

-

### Area

18611000

### Parts Per Million

0.5

### Fragmentation Mode

HCD
