# Supplementary material for: Template-Based Assembly of Proteomic Short Reads For De Novo Antibody Sequencing and Repertoire Profiling
Source: Anal Chem. 2022 Jul 14;94(29):10391–9. doi: 10.1021/acs.analchem.2c01300 (PMC9330293; doi:10.1021/acs.analchem.2c01300)
Supplement: Supplementary file 2 — ac2c01300_si_002.zip [file ac2c01300_si_002.zip › Schulte_2022_ACS-AC_Stitch_SupplementaryData/2022-06-22@17-20-24 anti-FLAG-M2/report-monoclonal/reads/F1_10484.html]

Details F1\_10484

OverviewUndefined

# Read F1:10484

## Sequence

DAGKAPCLCTVPEVSSVFLFPPKPK

## Sequence Length

25

## Meta Information from PEAKS

### Scan Identifier

F1:10484

### Original Sequence (length=41)

D

A

G

K

A

P

C

+58.01

L

C

+58.01

T

V

P

E

V

S

S

V

F

L

F

P

P

K

P

K

### Posttranslational Modifications

Carboxymethyl

### Source File

20191211\_F1\_Ag5\_peng0013\_SA\_Flag\_Asp\_N.raw

### Fraction

1

### Scan Feature

-

### De Novo Score

90

### Confidence score

90

### Mass Charge Ratio

687.3503

### Mass

2745.3757

### Charge

4

### Retention Time

58.15

### Predicted Retention Time

-

### Area

0

### Fragmentation Mode

HCD
