# Supplementary material for: Template-Based Assembly of Proteomic Short Reads For De Novo Antibody Sequencing and Repertoire Profiling
Source: Anal Chem. 2022 Jul 14;94(29):10391–9. doi: 10.1021/acs.analchem.2c01300 (PMC9330293; doi:10.1021/acs.analchem.2c01300)
Supplement: Supplementary file 2 — ac2c01300_si_002.zip [file ac2c01300_si_002.zip › Schulte_2022_ACS-AC_Stitch_SupplementaryData/2022-06-22@17-20-24 anti-FLAG-M2/report-monoclonal/reads/F1_10517.html]

Details F1\_10517

OverviewUndefined

# Read F1:10517

## Sequence

DFAELTKVKDLTKVNKECCHG

## Sequence Length

21

## Meta Information from PEAKS

### Scan Identifier

F1:10517

### Original Sequence (length=45)

D

F

A

E

L

T

K

V

K

+58.01

D

L

T

K

V

N

K

E

C

+58.01

C

+58.01

H

G

### Posttranslational Modifications

Carboxymethyl (KW X@N-term); Carboxymethyl

### Source File

20191211\_F1\_Ag5\_peng0013\_SA\_Flag\_Asp\_N.raw

### Fraction

1

### Scan Feature

F1:9887

### De Novo Score

93

### Confidence score

93

### Mass Charge Ratio

638.8051

### Mass

2551.1934

### Charge

4

### Retention Time

58.34

### Predicted Retention Time

-

### Area

2737500

### Fragmentation Mode

ETHCD
