# Supplementary material for: Template-Based Assembly of Proteomic Short Reads For De Novo Antibody Sequencing and Repertoire Profiling
Source: Anal Chem. 2022 Jul 14;94(29):10391–9. doi: 10.1021/acs.analchem.2c01300 (PMC9330293; doi:10.1021/acs.analchem.2c01300)
Supplement: Supplementary file 2 — ac2c01300_si_002.zip [file ac2c01300_si_002.zip › Schulte_2022_ACS-AC_Stitch_SupplementaryData/2022-06-22@17-20-24 anti-FLAG-M2/report-monoclonal/reads/F1_10529.html]

Details F1\_10529

OverviewUndefined

# Read F1:10529

## Sequence

DCGCKPCLCTVPEVSSVFLFPPKPK

## Sequence Length

25

## Meta Information from PEAKS

### Scan Identifier

F1:10529

### Original Sequence (length=57)

D

C

+58.01

G

C

+58.01

K

P

C

+58.01

L

C

+58.01

T

V

P

E

V

S

S

V

F

L

F

P

P

K

P

K

### Posttranslational Modifications

Carboxymethyl

### Source File

20191211\_F1\_Ag5\_peng0013\_SA\_Flag\_Asp\_N.raw

### Fraction

1

### Scan Feature

F1:13126

### De Novo Score

97

### Confidence score

97

### Mass Charge Ratio

732.3407

### Mass

2925.3308

### Charge

4

### Retention Time

58.4

### Predicted Retention Time

-

### Area

688030000

### Parts Per Million

1

### Fragmentation Mode

HCD

### Also found in scans

F1:10513 F1:10500
