# Supplementary material for: Template-Based Assembly of Proteomic Short Reads For De Novo Antibody Sequencing and Repertoire Profiling
Source: Anal Chem. 2022 Jul 14;94(29):10391–9. doi: 10.1021/acs.analchem.2c01300 (PMC9330293; doi:10.1021/acs.analchem.2c01300)
Supplement: Supplementary file 2 — ac2c01300_si_002.zip [file ac2c01300_si_002.zip › Schulte_2022_ACS-AC_Stitch_SupplementaryData/2022-06-22@17-20-24 anti-FLAG-M2/report-monoclonal/reads/F1_10734.html]

Details F1\_10734

OverviewUndefined

# Read F1:10734

## Sequence

DLGVYYCFQGSNHKTTFGGGTKL

## Sequence Length

23

## Meta Information from PEAKS

### Scan Identifier

F1:10734

### Original Sequence (length=47)

D

L

G

V

Y

Y

C

+58.01

F

Q

G

S

N

H

+15.99

K

+58.01

T

T

F

G

G

G

T

K

L

### Posttranslational Modifications

Carboxymethyl; Oxidation (HW); Carboxymethyl (KW X@N-term)

### Source File

20191211\_F1\_Ag5\_peng0013\_SA\_Flag\_Asp\_N.raw

### Fraction

1

### Scan Feature

F1:17621

### De Novo Score

91

### Confidence score

91

### Mass Charge Ratio

875.7373

### Mass

2624.1853

### Charge

3

### Retention Time

59.72

### Predicted Retention Time

-

### Area

2733000

### Parts Per Million

1.8

### Fragmentation Mode

ETHCD
