# Supplementary material for: Template-Based Assembly of Proteomic Short Reads For De Novo Antibody Sequencing and Repertoire Profiling
Source: Anal Chem. 2022 Jul 14;94(29):10391–9. doi: 10.1021/acs.analchem.2c01300 (PMC9330293; doi:10.1021/acs.analchem.2c01300)
Supplement: Supplementary file 2 — ac2c01300_si_002.zip [file ac2c01300_si_002.zip › Schulte_2022_ACS-AC_Stitch_SupplementaryData/2022-06-22@17-20-24 anti-FLAG-M2/report-monoclonal/reads/F1_10973.html]

Details F1\_10973

OverviewUndefined

# Read F1:10973

## Sequence

DLAWLGYLNPSSGYAAYNQNFK

## Sequence Length

22

## Meta Information from PEAKS

### Scan Identifier

F1:10973

### Original Sequence (length=22)

D

L

A

W

L

G

Y

L

N

P

S

S

G

Y

A

A

Y

N

Q

N

F

K

### Posttranslational Modifications

### Source File

20191211\_F1\_Ag5\_peng0013\_SA\_Flag\_Asp\_N.raw

### Fraction

1

### Scan Feature

F1:16246

### De Novo Score

94

### Confidence score

94

### Mass Charge Ratio

831.402

### Mass

2491.1807

### Charge

3

### Retention Time

60.97

### Predicted Retention Time

-

### Area

812440

### Parts Per Million

1.4

### Fragmentation Mode

HCD
