# Supplementary material for: Template-Based Assembly of Proteomic Short Reads For De Novo Antibody Sequencing and Repertoire Profiling
Source: Anal Chem. 2022 Jul 14;94(29):10391–9. doi: 10.1021/acs.analchem.2c01300 (PMC9330293; doi:10.1021/acs.analchem.2c01300)
Supplement: Supplementary file 2 — ac2c01300_si_002.zip [file ac2c01300_si_002.zip › Schulte_2022_ACS-AC_Stitch_SupplementaryData/2022-06-22@17-20-24 anti-FLAG-M2/report-monoclonal/reads/F1_11057.html]

Details F1\_11057

OverviewUndefined

# Read F1:11057

## Sequence

DVLTLTLTPKVTCVVV

## Sequence Length

16

## Meta Information from PEAKS

### Scan Identifier

F1:11057

### Original Sequence (length=24)

D

V

L

T

L

T

L

T

P

K

V

T

C

+58.01

V

V

V

### Posttranslational Modifications

Carboxymethyl

### Source File

20191211\_F1\_Ag5\_peng0013\_SA\_Flag\_Asp\_N.raw

### Fraction

1

### Scan Feature

F1:7578

### De Novo Score

98

### Confidence score

98

### Mass Charge Ratio

587.0016

### Mass

1757.9849

### Charge

3

### Retention Time

61.55

### Predicted Retention Time

-

### Area

83099000

### Fragmentation Mode

ETHCD

### Also found in scans

F1:11535 F1:11332 F1:11449 F1:11942 F1:11601 F1:12007 F1:11417 F1:11770 F1:11362 F1:11538 F1:13817 F1:12199 F1:11766 F1:11576
