# Supplementary material for: Template-Based Assembly of Proteomic Short Reads For De Novo Antibody Sequencing and Repertoire Profiling
Source: Anal Chem. 2022 Jul 14;94(29):10391–9. doi: 10.1021/acs.analchem.2c01300 (PMC9330293; doi:10.1021/acs.analchem.2c01300)
Supplement: Supplementary file 2 — ac2c01300_si_002.zip [file ac2c01300_si_002.zip › Schulte_2022_ACS-AC_Stitch_SupplementaryData/2022-06-22@17-20-24 anti-FLAG-M2/report-monoclonal/reads/F1_11458.html]

Details F1\_11458

OverviewUndefined

# Read F1:11458

## Sequence

DPSSSTAYMELGGSLTSEDSAVYYCAREKFYGY

## Sequence Length

33

## Meta Information from PEAKS

### Scan Identifier

F1:11458

### Original Sequence (length=49)

D

P

S

S

S

T

A

Y

M

+15.99

E

L

G

G

S

L

T

S

E

D

S

A

V

Y

Y

C

+58.01

A

R

E

K

F

Y

G

Y

### Posttranslational Modifications

Oxidation (M); Carboxymethyl

### Source File

20191211\_F1\_Ag5\_peng0013\_SA\_Flag\_Asp\_N.raw

### Fraction

1

### Scan Feature

F1:24241

### De Novo Score

99

### Confidence score

99

### Mass Charge Ratio

1241.2002

### Mass

3720.5759

### Charge

3

### Retention Time

63.73

### Predicted Retention Time

-

### Area

212970000

### Parts Per Million

0.8

### Fragmentation Mode

HCD
