# Supplementary material for: Template-Based Assembly of Proteomic Short Reads For De Novo Antibody Sequencing and Repertoire Profiling
Source: Anal Chem. 2022 Jul 14;94(29):10391–9. doi: 10.1021/acs.analchem.2c01300 (PMC9330293; doi:10.1021/acs.analchem.2c01300)
Supplement: Supplementary file 2 — ac2c01300_si_002.zip [file ac2c01300_si_002.zip › Schulte_2022_ACS-AC_Stitch_SupplementaryData/2022-06-22@17-20-24 anti-FLAG-M2/report-monoclonal/reads/F1_11597.html]

Details F1\_11597

OverviewUndefined

# Read F1:11597

## Sequence

DALNFLRALY

## Sequence Length

10

## Meta Information from PEAKS

### Scan Identifier

F1:11597

### Original Sequence (length=18)

D

+58.01

A

L

N

F

L

R

A

L

Y

### Posttranslational Modifications

Carboxymethyl (KW X@N-term)

### Source File

20191211\_F1\_Ag5\_peng0013\_SA\_Flag\_Asp\_N.raw

### Fraction

1

### Scan Feature

F1:9287

### De Novo Score

96

### Confidence score

96

### Mass Charge Ratio

627.3303

### Mass

1252.645

### Charge

2

### Retention Time

64.59

### Predicted Retention Time

-

### Area

394120

### Parts Per Million

0.9

### Fragmentation Mode

HCD
