# Supplementary material for: Template-Based Assembly of Proteomic Short Reads For De Novo Antibody Sequencing and Repertoire Profiling
Source: Anal Chem. 2022 Jul 14;94(29):10391–9. doi: 10.1021/acs.analchem.2c01300 (PMC9330293; doi:10.1021/acs.analchem.2c01300)
Supplement: Supplementary file 2 — ac2c01300_si_002.zip [file ac2c01300_si_002.zip › Schulte_2022_ACS-AC_Stitch_SupplementaryData/2022-06-22@17-20-24 anti-FLAG-M2/report-monoclonal/reads/F1_11691.html]

Details F1\_11691

OverviewUndefined

# Read F1:11691

## Sequence

DLSKDDPEVQFSMAFV

## Sequence Length

16

## Meta Information from PEAKS

### Scan Identifier

F1:11691

### Original Sequence (length=24)

D

L

S

K

D

D

P

E

V

Q

F

S

M

+15.99

A

F

V

### Posttranslational Modifications

Oxidation (M)

### Source File

20191211\_F1\_Ag5\_peng0013\_SA\_Flag\_Asp\_N.raw

### Fraction

1

### Scan Feature

F1:19168

### De Novo Score

91

### Confidence score

91

### Mass Charge Ratio

922.4226

### Mass

1842.8345

### Charge

2

### Retention Time

64.95

### Predicted Retention Time

-

### Area

7710900

### Fragmentation Mode

ETHCD
