# Supplementary material for: Template-Based Assembly of Proteomic Short Reads For De Novo Antibody Sequencing and Repertoire Profiling
Source: Anal Chem. 2022 Jul 14;94(29):10391–9. doi: 10.1021/acs.analchem.2c01300 (PMC9330293; doi:10.1021/acs.analchem.2c01300)
Supplement: Supplementary file 2 — ac2c01300_si_002.zip [file ac2c01300_si_002.zip › Schulte_2022_ACS-AC_Stitch_SupplementaryData/2022-06-22@17-20-24 anti-FLAG-M2/report-monoclonal/reads/F1_11705.html]

Details F1\_11705

OverviewUndefined

# Read F1:11705

## Sequence

DETTLTADPSSSTAYMELNSLTSEDSAVYYYTTDATLTDHG

## Sequence Length

41

## Meta Information from PEAKS

### Scan Identifier

F1:11705

### Original Sequence (length=49)

D

E

T

T

L

T

A

D

P

S

S

S

T

A

Y

M

+15.99

E

L

N

S

L

T

S

E

D

S

A

V

Y

Y

Y

T

T

D

A

T

L

T

D

H

G

### Posttranslational Modifications

Oxidation (M)

### Source File

20191211\_F1\_Ag5\_peng0013\_SA\_Flag\_Asp\_N.raw

### Fraction

1

### Scan Feature

F1:25283

### De Novo Score

98

### Confidence score

98

### Mass Charge Ratio

1484.9763

### Mass

4451.9126

### Charge

3

### Retention Time

65.31

### Predicted Retention Time

-

### Area

42575000

### Fragmentation Mode

HCD
