# Supplementary material for: Template-Based Assembly of Proteomic Short Reads For De Novo Antibody Sequencing and Repertoire Profiling
Source: Anal Chem. 2022 Jul 14;94(29):10391–9. doi: 10.1021/acs.analchem.2c01300 (PMC9330293; doi:10.1021/acs.analchem.2c01300)
Supplement: Supplementary file 2 — ac2c01300_si_002.zip [file ac2c01300_si_002.zip › Schulte_2022_ACS-AC_Stitch_SupplementaryData/2022-06-22@17-20-24 anti-FLAG-M2/report-monoclonal/reads/F1_11719.html]

Details F1\_11719

OverviewUndefined

# Read F1:11719

## Sequence

DLKAFLNNFYPK

## Sequence Length

12

## Meta Information from PEAKS

### Scan Identifier

F1:11719

### Original Sequence (length=12)

D

L

K

A

F

L

N

N

F

Y

P

K

### Posttranslational Modifications

### Source File

20191211\_F1\_Ag5\_peng0013\_SA\_Flag\_Asp\_N.raw

### Fraction

1

### Scan Feature

F1:13224

### De Novo Score

93

### Confidence score

93

### Mass Charge Ratio

735.3948

### Mass

1468.7715

### Charge

2

### Retention Time

65.26

### Predicted Retention Time

-

### Area

158790

### Parts Per Million

2.4

### Fragmentation Mode

HCD
