# Supplementary material for: Template-Based Assembly of Proteomic Short Reads For De Novo Antibody Sequencing and Repertoire Profiling
Source: Anal Chem. 2022 Jul 14;94(29):10391–9. doi: 10.1021/acs.analchem.2c01300 (PMC9330293; doi:10.1021/acs.analchem.2c01300)
Supplement: Supplementary file 2 — ac2c01300_si_002.zip [file ac2c01300_si_002.zip › Schulte_2022_ACS-AC_Stitch_SupplementaryData/2022-06-22@17-20-24 anti-FLAG-M2/report-monoclonal/reads/F1_11750.html]

Details F1\_11750

OverviewUndefined

# Read F1:11750

## Sequence

DPHTNATYVQFSWFVDDVEVH

## Sequence Length

21

## Meta Information from PEAKS

### Scan Identifier

F1:11750

### Original Sequence (length=21)

D

P

H

T

N

A

T

Y

V

Q

F

S

W

F

V

D

D

V

E

V

H

### Posttranslational Modifications

### Source File

20191211\_F1\_Ag5\_peng0013\_SA\_Flag\_Asp\_N.raw

### Fraction

1

### Scan Feature

F1:16438

### De Novo Score

92

### Confidence score

92

### Mass Charge Ratio

836.0504

### Mass

2505.1235

### Charge

3

### Retention Time

65.48

### Predicted Retention Time

-

### Area

195350

### Parts Per Million

2.3

### Fragmentation Mode

ETHCD
