# Supplementary material for: Template-Based Assembly of Proteomic Short Reads For De Novo Antibody Sequencing and Repertoire Profiling
Source: Anal Chem. 2022 Jul 14;94(29):10391–9. doi: 10.1021/acs.analchem.2c01300 (PMC9330293; doi:10.1021/acs.analchem.2c01300)
Supplement: Supplementary file 2 — ac2c01300_si_002.zip [file ac2c01300_si_002.zip › Schulte_2022_ACS-AC_Stitch_SupplementaryData/2022-06-22@17-20-24 anti-FLAG-M2/report-monoclonal/reads/F1_11757.html]

Details F1\_11757

OverviewUndefined

# Read F1:11757

## Sequence

DATTLTADPSSSTAYMELNSLTSWASVYYYWDATLTDGH

## Sequence Length

39

## Meta Information from PEAKS

### Scan Identifier

F1:11757

### Original Sequence (length=71)

D

+58.01

A

T

T

L

T

A

D

P

S

S

S

T

A

Y

M

+15.99

E

L

N

S

L

T

S

W

+58.01

A

S

V

Y

Y

Y

W

+15.99

D

A

T

L

T

D

G

H

### Posttranslational Modifications

Carboxymethyl (KW X@N-term); Oxidation (M); Oxidation (HW)

### Source File

20191211\_F1\_Ag5\_peng0013\_SA\_Flag\_Asp\_N.raw

### Fraction

1

### Scan Feature

F1:22501

### De Novo Score

94

### Confidence score

94

### Mass Charge Ratio

1113.9844

### Mass

4451.9067

### Charge

4

### Retention Time

65.31

### Predicted Retention Time

-

### Area

11989000

### Parts Per Million

0.4

### Fragmentation Mode

HCD
