# Supplementary material for: Template-Based Assembly of Proteomic Short Reads For De Novo Antibody Sequencing and Repertoire Profiling
Source: Anal Chem. 2022 Jul 14;94(29):10391–9. doi: 10.1021/acs.analchem.2c01300 (PMC9330293; doi:10.1021/acs.analchem.2c01300)
Supplement: Supplementary file 2 — ac2c01300_si_002.zip [file ac2c01300_si_002.zip › Schulte_2022_ACS-AC_Stitch_SupplementaryData/2022-06-22@17-20-24 anti-FLAG-M2/report-monoclonal/reads/F1_11803.html]

Details F1\_11803

OverviewUndefined

# Read F1:11803

## Sequence

DKNYDDLTVEWQWNGQPAENYKNTPQLM

## Sequence Length

28

## Meta Information from PEAKS

### Scan Identifier

F1:11803

### Original Sequence (length=36)

D

K

N

Y

D

D

L

T

V

E

W

Q

W

N

G

Q

P

A

E

N

Y

K

N

T

P

Q

L

M

+15.99

### Posttranslational Modifications

Oxidation (M)

### Source File

20191211\_F1\_Ag5\_peng0013\_SA\_Flag\_Asp\_N.raw

### Fraction

1

### Scan Feature

F1:22918

### De Novo Score

92

### Confidence score

92

### Mass Charge Ratio

1138.5183

### Mass

3412.5305

### Charge

3

### Retention Time

65.82

### Predicted Retention Time

-

### Area

28683000

### Parts Per Million

0.8

### Fragmentation Mode

ETHCD
