# Supplementary material for: Template-Based Assembly of Proteomic Short Reads For De Novo Antibody Sequencing and Repertoire Profiling
Source: Anal Chem. 2022 Jul 14;94(29):10391–9. doi: 10.1021/acs.analchem.2c01300 (PMC9330293; doi:10.1021/acs.analchem.2c01300)
Supplement: Supplementary file 2 — ac2c01300_si_002.zip [file ac2c01300_si_002.zip › Schulte_2022_ACS-AC_Stitch_SupplementaryData/2022-06-22@17-20-24 anti-FLAG-M2/report-monoclonal/reads/F1_11824.html]

Details F1\_11824

OverviewUndefined

# Read F1:11824

## Sequence

DVLTPNLPLSLPVSLG

## Sequence Length

16

## Meta Information from PEAKS

### Scan Identifier

F1:11824

### Original Sequence (length=16)

D

V

L

T

P

N

L

P

L

S

L

P

V

S

L

G

### Posttranslational Modifications

### Source File

20191211\_F1\_Ag5\_peng0013\_SA\_Flag\_Asp\_N.raw

### Fraction

1

### Scan Feature

F1:6226

### De Novo Score

91

### Confidence score

91

### Mass Charge Ratio

545.6503

### Mass

1633.929

### Charge

3

### Retention Time

65.94

### Predicted Retention Time

-

### Area

11095000

### Parts Per Million

0

### Fragmentation Mode

ETHCD
