# Supplementary material for: Template-Based Assembly of Proteomic Short Reads For De Novo Antibody Sequencing and Repertoire Profiling
Source: Anal Chem. 2022 Jul 14;94(29):10391–9. doi: 10.1021/acs.analchem.2c01300 (PMC9330293; doi:10.1021/acs.analchem.2c01300)
Supplement: Supplementary file 2 — ac2c01300_si_002.zip [file ac2c01300_si_002.zip › Schulte_2022_ACS-AC_Stitch_SupplementaryData/2022-06-22@17-20-24 anti-FLAG-M2/report-monoclonal/reads/F1_11905.html]

Details F1\_11905

OverviewUndefined

# Read F1:11905

## Sequence

DVVVCFLNNFFLAG

## Sequence Length

14

## Meta Information from PEAKS

### Scan Identifier

F1:11905

### Original Sequence (length=30)

D

+58.01

V

V

V

C

+58.01

F

L

N

N

F

F

L

A

G

### Posttranslational Modifications

Carboxymethyl (KW X@N-term); Carboxymethyl

### Source File

20191211\_F1\_Ag5\_peng0013\_SA\_Flag\_Asp\_N.raw

### Fraction

1

### Scan Feature

F1:16464

### De Novo Score

91

### Confidence score

91

### Mass Charge Ratio

837.4034

### Mass

1672.7808

### Charge

2

### Retention Time

66.58

### Predicted Retention Time

-

### Area

788410

### Parts Per Million

6.9

### Fragmentation Mode

ETHCD
