# Supplementary material for: Template-Based Assembly of Proteomic Short Reads For De Novo Antibody Sequencing and Repertoire Profiling
Source: Anal Chem. 2022 Jul 14;94(29):10391–9. doi: 10.1021/acs.analchem.2c01300 (PMC9330293; doi:10.1021/acs.analchem.2c01300)
Supplement: Supplementary file 2 — ac2c01300_si_002.zip [file ac2c01300_si_002.zip › Schulte_2022_ACS-AC_Stitch_SupplementaryData/2022-06-22@17-20-24 anti-FLAG-M2/report-monoclonal/reads/F1_11913.html]

Details F1\_11913

OverviewUndefined

# Read F1:11913

## Sequence

DVLTLTKGTLTLTWVV

## Sequence Length

16

## Meta Information from PEAKS

### Scan Identifier

F1:11913

### Original Sequence (length=32)

D

+58.01

V

L

T

L

T

K

G

T

L

T

L

T

W

+15.99

V

V

### Posttranslational Modifications

Carboxymethyl (KW X@N-term); Oxidation (HW)

### Source File

20191211\_F1\_Ag5\_peng0013\_SA\_Flag\_Asp\_N.raw

### Fraction

1

### Scan Feature

F1:18978

### De Novo Score

92

### Confidence score

92

### Mass Charge Ratio

917.5142

### Mass

1833.0134

### Charge

2

### Retention Time

66.43

### Predicted Retention Time

-

### Area

1119400

### Parts Per Million

0.2

### Fragmentation Mode

ETHCD
