# Supplementary material for: Template-Based Assembly of Proteomic Short Reads For De Novo Antibody Sequencing and Repertoire Profiling
Source: Anal Chem. 2022 Jul 14;94(29):10391–9. doi: 10.1021/acs.analchem.2c01300 (PMC9330293; doi:10.1021/acs.analchem.2c01300)
Supplement: Supplementary file 2 — ac2c01300_si_002.zip [file ac2c01300_si_002.zip › Schulte_2022_ACS-AC_Stitch_SupplementaryData/2022-06-22@17-20-24 anti-FLAG-M2/report-monoclonal/reads/F1_11933.html]

Details F1\_11933

OverviewUndefined

# Read F1:11933

## Sequence

DKLMTQLPLSLPVSLG

## Sequence Length

16

## Meta Information from PEAKS

### Scan Identifier

F1:11933

### Original Sequence (length=32)

D

K

+58.01

L

M

+15.99

T

Q

L

P

L

S

L

P

V

S

L

G

### Posttranslational Modifications

Carboxymethyl (KW X@N-term); Oxidation (M)

### Source File

20191211\_F1\_Ag5\_peng0013\_SA\_Flag\_Asp\_N.raw

### Fraction

1

### Scan Feature

F1:18142

### De Novo Score

97

### Confidence score

97

### Mass Charge Ratio

893.4874

### Mass

1784.9595

### Charge

2

### Retention Time

66.69

### Predicted Retention Time

-

### Area

24392000

### Parts Per Million

0.5

### Fragmentation Mode

ETHCD
