# Supplementary material for: Template-Based Assembly of Proteomic Short Reads For De Novo Antibody Sequencing and Repertoire Profiling
Source: Anal Chem. 2022 Jul 14;94(29):10391–9. doi: 10.1021/acs.analchem.2c01300 (PMC9330293; doi:10.1021/acs.analchem.2c01300)
Supplement: Supplementary file 2 — ac2c01300_si_002.zip [file ac2c01300_si_002.zip › Schulte_2022_ACS-AC_Stitch_SupplementaryData/2022-06-22@17-20-24 anti-FLAG-M2/report-monoclonal/reads/F1_12230.html]

Details F1\_12230

OverviewUndefined

# Read F1:12230

## Sequence

DLSKDDPEVQFSWFV

## Sequence Length

15

## Meta Information from PEAKS

### Scan Identifier

F1:12230

### Original Sequence (length=15)

D

L

S

K

D

D

P

E

V

Q

F

S

W

F

V

### Posttranslational Modifications

### Source File

20191211\_F1\_Ag5\_peng0013\_SA\_Flag\_Asp\_N.raw

### Fraction

1

### Scan Feature

F1:8207

### De Novo Score

99

### Confidence score

99

### Mass Charge Ratio

604.6203

### Mass

1810.8413

### Charge

3

### Retention Time

68.29

### Predicted Retention Time

-

### Area

24833000

### Fragmentation Mode

ETHCD

### Also found in scans

F1:11949 F1:12340 F1:11454 F1:12450 F1:12485
