# Supplementary material for: Template-Based Assembly of Proteomic Short Reads For De Novo Antibody Sequencing and Repertoire Profiling
Source: Anal Chem. 2022 Jul 14;94(29):10391–9. doi: 10.1021/acs.analchem.2c01300 (PMC9330293; doi:10.1021/acs.analchem.2c01300)
Supplement: Supplementary file 2 — ac2c01300_si_002.zip [file ac2c01300_si_002.zip › Schulte_2022_ACS-AC_Stitch_SupplementaryData/2022-06-22@17-20-24 anti-FLAG-M2/report-monoclonal/reads/F1_12451.html]

Details F1\_12451

OverviewUndefined

# Read F1:12451

## Sequence

DAALTSGGASVVCFLNNFYPK

## Sequence Length

21

## Meta Information from PEAKS

### Scan Identifier

F1:12451

### Original Sequence (length=29)

D

A

A

L

T

S

G

G

A

S

V

V

C

+58.01

F

L

N

N

F

Y

P

K

### Posttranslational Modifications

Carboxymethyl

### Source File

20191211\_F1\_Ag5\_peng0013\_SA\_Flag\_Asp\_N.raw

### Fraction

1

### Scan Feature

F1:22531

### De Novo Score

99

### Confidence score

99

### Mass Charge Ratio

1116.5349

### Mass

2231.0569

### Charge

2

### Retention Time

69.54

### Predicted Retention Time

-

### Area

1860000

### Fragmentation Mode

HCD

### Also found in scans

F1:12510
