# Supplementary material for: Template-Based Assembly of Proteomic Short Reads For De Novo Antibody Sequencing and Repertoire Profiling
Source: Anal Chem. 2022 Jul 14;94(29):10391–9. doi: 10.1021/acs.analchem.2c01300 (PMC9330293; doi:10.1021/acs.analchem.2c01300)
Supplement: Supplementary file 2 — ac2c01300_si_002.zip [file ac2c01300_si_002.zip › Schulte_2022_ACS-AC_Stitch_SupplementaryData/2022-06-22@17-20-24 anti-FLAG-M2/report-monoclonal/reads/F1_12463.html]

Details F1\_12463

OverviewUndefined

# Read F1:12463

## Sequence

DTQLTSNASVVCFLNNFYPK

## Sequence Length

20

## Meta Information from PEAKS

### Scan Identifier

F1:12463

### Original Sequence (length=28)

D

T

Q

L

T

S

N

A

S

V

V

C

+58.01

F

L

N

N

F

Y

P

K

### Posttranslational Modifications

Carboxymethyl

### Source File

20191211\_F1\_Ag5\_peng0013\_SA\_Flag\_Asp\_N.raw

### Fraction

1

### Scan Feature

F1:23177

### De Novo Score

94

### Confidence score

94

### Mass Charge Ratio

1160.0519

### Mass

2318.0889

### Charge

2

### Retention Time

69.54

### Predicted Retention Time

-

### Area

170270

### Parts Per Million

0.1

### Fragmentation Mode

HCD
