# Supplementary material for: Template-Based Assembly of Proteomic Short Reads For De Novo Antibody Sequencing and Repertoire Profiling
Source: Anal Chem. 2022 Jul 14;94(29):10391–9. doi: 10.1021/acs.analchem.2c01300 (PMC9330293; doi:10.1021/acs.analchem.2c01300)
Supplement: Supplementary file 2 — ac2c01300_si_002.zip [file ac2c01300_si_002.zip › Schulte_2022_ACS-AC_Stitch_SupplementaryData/2022-06-22@17-20-24 anti-FLAG-M2/report-monoclonal/reads/F1_13158.html]

Details F1\_13158

OverviewUndefined

# Read F1:13158

## Sequence

DAAPTVSLFPPSSEQLTSGGASVVCFL

## Sequence Length

27

## Meta Information from PEAKS

### Scan Identifier

F1:13158

### Original Sequence (length=35)

D

A

A

P

T

V

S

L

F

P

P

S

S

E

Q

L

T

S

G

G

A

S

V

V

C

+58.01

F

L

### Posttranslational Modifications

Carboxymethyl

### Source File

20191211\_F1\_Ag5\_peng0013\_SA\_Flag\_Asp\_N.raw

### Fraction

1

### Scan Feature

F1:18828

### De Novo Score

98

### Confidence score

98

### Mass Charge Ratio

913.4458

### Mass

2737.3154

### Charge

3

### Retention Time

73.32

### Predicted Retention Time

-

### Area

32001000

### Parts Per Million

0.1

### Fragmentation Mode

ETHCD
