# Supplementary material for: Template-Based Assembly of Proteomic Short Reads For De Novo Antibody Sequencing and Repertoire Profiling
Source: Anal Chem. 2022 Jul 14;94(29):10391–9. doi: 10.1021/acs.analchem.2c01300 (PMC9330293; doi:10.1021/acs.analchem.2c01300)
Supplement: Supplementary file 2 — ac2c01300_si_002.zip [file ac2c01300_si_002.zip › Schulte_2022_ACS-AC_Stitch_SupplementaryData/2022-06-22@17-20-24 anti-FLAG-M2/report-monoclonal/reads/F1_13652.html]

Details F1\_13652

OverviewUndefined

# Read F1:13652

## Sequence

DVLMTQLPLSLVPSLG

## Sequence Length

16

## Meta Information from PEAKS

### Scan Identifier

F1:13652

### Original Sequence (length=24)

D

+58.01

V

L

M

T

Q

L

P

L

S

L

V

P

S

L

G

### Posttranslational Modifications

Carboxymethyl (KW X@N-term)

### Source File

20191211\_F1\_Ag5\_peng0013\_SA\_Flag\_Asp\_N.raw

### Fraction

1

### Scan Feature

F1:17461

### De Novo Score

93

### Confidence score

93

### Mass Charge Ratio

870.9761

### Mass

1739.938

### Charge

2

### Retention Time

76.6

### Predicted Retention Time

-

### Area

3824500

### Fragmentation Mode

ETHCD
