# Supplementary material for: Template-Based Assembly of Proteomic Short Reads For De Novo Antibody Sequencing and Repertoire Profiling
Source: Anal Chem. 2022 Jul 14;94(29):10391–9. doi: 10.1021/acs.analchem.2c01300 (PMC9330293; doi:10.1021/acs.analchem.2c01300)
Supplement: Supplementary file 2 — ac2c01300_si_002.zip [file ac2c01300_si_002.zip › Schulte_2022_ACS-AC_Stitch_SupplementaryData/2022-06-22@17-20-24 anti-FLAG-M2/report-monoclonal/reads/F1_14239.html]

Details F1\_14239

OverviewUndefined

# Read F1:14239

## Sequence

DDPEVQFSWFV

## Sequence Length

11

## Meta Information from PEAKS

### Scan Identifier

F1:14239

### Original Sequence (length=11)

D

D

P

E

V

Q

F

S

W

F

V

### Posttranslational Modifications

### Source File

20191211\_F1\_Ag5\_peng0013\_SA\_Flag\_Asp\_N.raw

### Fraction

1

### Scan Feature

F1:11465

### De Novo Score

99

### Confidence score

99

### Mass Charge Ratio

684.8092

### Mass

1367.6033

### Charge

2

### Retention Time

79.54

### Predicted Retention Time

-

### Area

761160

### Parts Per Million

0.4

### Fragmentation Mode

ETHCD

### Also found in scans

F1:12643 F1:11889 F1:12992 F1:13241 F1:13305 F1:12590 F1:12135 F1:11827 F1:13360
