# Supplementary material for: Template-Based Assembly of Proteomic Short Reads For De Novo Antibody Sequencing and Repertoire Profiling
Source: Anal Chem. 2022 Jul 14;94(29):10391–9. doi: 10.1021/acs.analchem.2c01300 (PMC9330293; doi:10.1021/acs.analchem.2c01300)
Supplement: Supplementary file 2 — ac2c01300_si_002.zip [file ac2c01300_si_002.zip › Schulte_2022_ACS-AC_Stitch_SupplementaryData/2022-06-22@17-20-24 anti-FLAG-M2/report-monoclonal/reads/F1_14240.html]

Details F1\_14240

OverviewUndefined

# Read F1:14240

## Sequence

DVVAFLNNFYPK

## Sequence Length

12

## Meta Information from PEAKS

### Scan Identifier

F1:14240

### Original Sequence (length=12)

D

V

V

A

F

L

N

N

F

Y

P

K

### Posttranslational Modifications

### Source File

20191211\_F1\_Ag5\_peng0013\_SA\_Flag\_Asp\_N.raw

### Fraction

1

### Scan Feature

F1:12554

### De Novo Score

98

### Confidence score

98

### Mass Charge Ratio

713.8724

### Mass

1425.7292

### Charge

2

### Retention Time

79.53

### Predicted Retention Time

-

### Area

816100

### Parts Per Million

0.8

### Fragmentation Mode

ETHCD
