# Supplementary material for: Template-Based Assembly of Proteomic Short Reads For De Novo Antibody Sequencing and Repertoire Profiling
Source: Anal Chem. 2022 Jul 14;94(29):10391–9. doi: 10.1021/acs.analchem.2c01300 (PMC9330293; doi:10.1021/acs.analchem.2c01300)
Supplement: Supplementary file 2 — ac2c01300_si_002.zip [file ac2c01300_si_002.zip › Schulte_2022_ACS-AC_Stitch_SupplementaryData/2022-06-22@17-20-24 anti-FLAG-M2/report-monoclonal/reads/F1_3451.html]

Details F1\_3451

OverviewUndefined

# Read F1:3451

## Sequence

DVVPPGLHNHHTEKSLSHSPG

## Sequence Length

21

## Meta Information from PEAKS

### Scan Identifier

F1:3451

### Original Sequence (length=29)

D

+58.01

V

V

P

P

G

L

H

N

H

H

T

E

K

S

L

S

H

S

P

G

### Posttranslational Modifications

Carboxymethyl (KW X@N-term)

### Source File

20191211\_F1\_Ag5\_peng0013\_SA\_Flag\_Asp\_N.raw

### Fraction

1

### Scan Feature

F1:7226

### De Novo Score

90

### Confidence score

90

### Mass Charge Ratio

576.5377

### Mass

2302.1089

### Charge

4

### Retention Time

18.76

### Predicted Retention Time

-

### Area

578590

### Parts Per Million

5.6

### Fragmentation Mode

ETHCD
