# Supplementary material for: Template-Based Assembly of Proteomic Short Reads For De Novo Antibody Sequencing and Repertoire Profiling
Source: Anal Chem. 2022 Jul 14;94(29):10391–9. doi: 10.1021/acs.analchem.2c01300 (PMC9330293; doi:10.1021/acs.analchem.2c01300)
Supplement: Supplementary file 2 — ac2c01300_si_002.zip [file ac2c01300_si_002.zip › Schulte_2022_ACS-AC_Stitch_SupplementaryData/2022-06-22@17-20-24 anti-FLAG-M2/report-monoclonal/reads/F1_3596.html]

Details F1\_3596

OverviewUndefined

# Read F1:3596

## Sequence

DDVEVHTAQTQPR

## Sequence Length

13

## Meta Information from PEAKS

### Scan Identifier

F1:3596

### Original Sequence (length=13)

D

D

V

E

V

H

T

A

Q

T

Q

P

R

### Posttranslational Modifications

### Source File

20191211\_F1\_Ag5\_peng0013\_SA\_Flag\_Asp\_N.raw

### Fraction

1

### Scan Feature

F1:4956

### De Novo Score

98

### Confidence score

98

### Mass Charge Ratio

499.2432

### Mass

1494.7063

### Charge

3

### Retention Time

19.56

### Predicted Retention Time

-

### Area

15663000

### Parts Per Million

1

### Fragmentation Mode

HCD

### Also found in scans

F1:3626
