# Supplementary material for: Template-Based Assembly of Proteomic Short Reads For De Novo Antibody Sequencing and Repertoire Profiling
Source: Anal Chem. 2022 Jul 14;94(29):10391–9. doi: 10.1021/acs.analchem.2c01300 (PMC9330293; doi:10.1021/acs.analchem.2c01300)
Supplement: Supplementary file 2 — ac2c01300_si_002.zip [file ac2c01300_si_002.zip › Schulte_2022_ACS-AC_Stitch_SupplementaryData/2022-06-22@17-20-24 anti-FLAG-M2/report-monoclonal/reads/F1_3616.html]

Details F1\_3616

OverviewUndefined

# Read F1:3616

## Sequence

DKGGNGGGLGSGGTVRSS

## Sequence Length

18

## Meta Information from PEAKS

### Scan Identifier

F1:3616

### Original Sequence (length=18)

D

K

G

G

N

G

G

G

L

G

S

G

G

T

V

R

S

S

### Posttranslational Modifications

### Source File

20191211\_F1\_Ag5\_peng0013\_SA\_Flag\_Asp\_N.raw

### Fraction

1

### Scan Feature

F1:5528

### De Novo Score

94

### Confidence score

94

### Mass Charge Ratio

521.5928

### Mass

1561.7444

### Charge

3

### Retention Time

19.72

### Predicted Retention Time

-

### Area

53587

### Parts Per Million

7.8

### Fragmentation Mode

ETHCD
