# Supplementary material for: Template-Based Assembly of Proteomic Short Reads For De Novo Antibody Sequencing and Repertoire Profiling
Source: Anal Chem. 2022 Jul 14;94(29):10391–9. doi: 10.1021/acs.analchem.2c01300 (PMC9330293; doi:10.1021/acs.analchem.2c01300)
Supplement: Supplementary file 2 — ac2c01300_si_002.zip [file ac2c01300_si_002.zip › Schulte_2022_ACS-AC_Stitch_SupplementaryData/2022-06-22@17-20-24 anti-FLAG-M2/report-monoclonal/reads/F1_3639.html]

Details F1\_3639

OverviewUndefined

# Read F1:3639

## Sequence

DLSGSPATLR

## Sequence Length

10

## Meta Information from PEAKS

### Scan Identifier

F1:3639

### Original Sequence (length=18)

D

+58.01

L

S

G

S

P

A

T

L

R

### Posttranslational Modifications

Carboxymethyl (KW X@N-term)

### Source File

20191211\_F1\_Ag5\_peng0013\_SA\_Flag\_Asp\_N.raw

### Fraction

1

### Scan Feature

F1:6013

### De Novo Score

93

### Confidence score

93

### Mass Charge Ratio

537.7804

### Mass

1073.5352

### Charge

2

### Retention Time

19.84

### Predicted Retention Time

-

### Area

1542400

### Parts Per Million

10.3

### Fragmentation Mode

HCD
