# Supplementary material for: Template-Based Assembly of Proteomic Short Reads For De Novo Antibody Sequencing and Repertoire Profiling
Source: Anal Chem. 2022 Jul 14;94(29):10391–9. doi: 10.1021/acs.analchem.2c01300 (PMC9330293; doi:10.1021/acs.analchem.2c01300)
Supplement: Supplementary file 2 — ac2c01300_si_002.zip [file ac2c01300_si_002.zip › Schulte_2022_ACS-AC_Stitch_SupplementaryData/2022-06-22@17-20-24 anti-FLAG-M2/report-monoclonal/reads/F1_3681.html]

Details F1\_3681

OverviewUndefined

# Read F1:3681

## Sequence

DAALKTVM

## Sequence Length

8

## Meta Information from PEAKS

### Scan Identifier

F1:3681

### Original Sequence (length=16)

D

A

A

L

K

T

V

M

+15.99

### Posttranslational Modifications

Oxidation (M)

### Source File

20191211\_F1\_Ag5\_peng0013\_SA\_Flag\_Asp\_N.raw

### Fraction

1

### Scan Feature

F1:3022

### De Novo Score

92

### Confidence score

92

### Mass Charge Ratio

432.7287

### Mass

863.4423

### Charge

2

### Retention Time

20.04

### Predicted Retention Time

-

### Area

228070

### Parts Per Million

0.6

### Fragmentation Mode

HCD
