# Supplementary material for: Template-Based Assembly of Proteomic Short Reads For De Novo Antibody Sequencing and Repertoire Profiling
Source: Anal Chem. 2022 Jul 14;94(29):10391–9. doi: 10.1021/acs.analchem.2c01300 (PMC9330293; doi:10.1021/acs.analchem.2c01300)
Supplement: Supplementary file 2 — ac2c01300_si_002.zip [file ac2c01300_si_002.zip › Schulte_2022_ACS-AC_Stitch_SupplementaryData/2022-06-22@17-20-24 anti-FLAG-M2/report-monoclonal/reads/F1_3720.html]

Details F1\_3720

OverviewUndefined

# Read F1:3720

## Sequence

DDVEVHTAQTQPREE

## Sequence Length

15

## Meta Information from PEAKS

### Scan Identifier

F1:3720

### Original Sequence (length=15)

D

D

V

E

V

H

T

A

Q

T

Q

P

R

E

E

### Posttranslational Modifications

### Source File

20191211\_F1\_Ag5\_peng0013\_SA\_Flag\_Asp\_N.raw

### Fraction

1

### Scan Feature

F1:7522

### De Novo Score

91

### Confidence score

91

### Mass Charge Ratio

585.27

### Mass

1752.7915

### Charge

3

### Retention Time

20.27

### Predicted Retention Time

-

### Area

17693

### Fragmentation Mode

ETHCD
