# Supplementary material for: Template-Based Assembly of Proteomic Short Reads For De Novo Antibody Sequencing and Repertoire Profiling
Source: Anal Chem. 2022 Jul 14;94(29):10391–9. doi: 10.1021/acs.analchem.2c01300 (PMC9330293; doi:10.1021/acs.analchem.2c01300)
Supplement: Supplementary file 2 — ac2c01300_si_002.zip [file ac2c01300_si_002.zip › Schulte_2022_ACS-AC_Stitch_SupplementaryData/2022-06-22@17-20-24 anti-FLAG-M2/report-monoclonal/reads/F1_3751.html]

Details F1\_3751

OverviewUndefined

# Read F1:3751

## Sequence

DNQRVLVNTK

## Sequence Length

10

## Meta Information from PEAKS

### Scan Identifier

F1:3751

### Original Sequence (length=10)

D

N

Q

R

V

L

V

N

T

K

### Posttranslational Modifications

### Source File

20191211\_F1\_Ag5\_peng0013\_SA\_Flag\_Asp\_N.raw

### Fraction

1

### Scan Feature

F1:1601

### De Novo Score

98

### Confidence score

98

### Mass Charge Ratio

396.2229

### Mass

1185.6465

### Charge

3

### Retention Time

20.47

### Predicted Retention Time

-

### Area

907250

### Parts Per Million

0.4

### Fragmentation Mode

HCD
