# Supplementary material for: Template-Based Assembly of Proteomic Short Reads For De Novo Antibody Sequencing and Repertoire Profiling
Source: Anal Chem. 2022 Jul 14;94(29):10391–9. doi: 10.1021/acs.analchem.2c01300 (PMC9330293; doi:10.1021/acs.analchem.2c01300)
Supplement: Supplementary file 2 — ac2c01300_si_002.zip [file ac2c01300_si_002.zip › Schulte_2022_ACS-AC_Stitch_SupplementaryData/2022-06-22@17-20-24 anti-FLAG-M2/report-monoclonal/reads/F1_3807.html]

Details F1\_3807

OverviewUndefined

# Read F1:3807

## Sequence

DTFSHEGLHNHHTEKSLSHSPG

## Sequence Length

22

## Meta Information from PEAKS

### Scan Identifier

F1:3807

### Original Sequence (length=22)

D

T

F

S

H

E

G

L

H

N

H

H

T

E

K

S

L

S

H

S

P

G

### Posttranslational Modifications

### Source File

20191211\_F1\_Ag5\_peng0013\_SA\_Flag\_Asp\_N.raw

### Fraction

1

### Scan Feature

F1:8609

### De Novo Score

91

### Confidence score

91

### Mass Charge Ratio

614.2861

### Mass

2453.1108

### Charge

4

### Retention Time

20.78

### Predicted Retention Time

-

### Area

1826200

### Parts Per Million

1.9

### Fragmentation Mode

ETHCD
