# Supplementary material for: Template-Based Assembly of Proteomic Short Reads For De Novo Antibody Sequencing and Repertoire Profiling
Source: Anal Chem. 2022 Jul 14;94(29):10391–9. doi: 10.1021/acs.analchem.2c01300 (PMC9330293; doi:10.1021/acs.analchem.2c01300)
Supplement: Supplementary file 2 — ac2c01300_si_002.zip [file ac2c01300_si_002.zip › Schulte_2022_ACS-AC_Stitch_SupplementaryData/2022-06-22@17-20-24 anti-FLAG-M2/report-monoclonal/reads/F1_3832.html]

Details F1\_3832

OverviewUndefined

# Read F1:3832

## Sequence

DEYERHNSYTC

## Sequence Length

11

## Meta Information from PEAKS

### Scan Identifier

F1:3832

### Original Sequence (length=19)

D

E

Y

E

R

H

N

S

Y

T

C

+58.01

### Posttranslational Modifications

Carboxymethyl

### Source File

20191211\_F1\_Ag5\_peng0013\_SA\_Flag\_Asp\_N.raw

### Fraction

1

### Scan Feature

F1:4771

### De Novo Score

99

### Confidence score

99

### Mass Charge Ratio

492.1901

### Mass

1473.5466

### Charge

3

### Retention Time

20.93

### Predicted Retention Time

-

### Area

3246200

### Parts Per Million

1.2

### Fragmentation Mode

ETHCD

### Also found in scans

F1:3843
