# Supplementary material for: Template-Based Assembly of Proteomic Short Reads For De Novo Antibody Sequencing and Repertoire Profiling
Source: Anal Chem. 2022 Jul 14;94(29):10391–9. doi: 10.1021/acs.analchem.2c01300 (PMC9330293; doi:10.1021/acs.analchem.2c01300)
Supplement: Supplementary file 2 — ac2c01300_si_002.zip [file ac2c01300_si_002.zip › Schulte_2022_ACS-AC_Stitch_SupplementaryData/2022-06-22@17-20-24 anti-FLAG-M2/report-monoclonal/reads/F1_3850.html]

Details F1\_3850

OverviewUndefined

# Read F1:3850

## Sequence

DPPKTSTSPLVKSFNRNQ

## Sequence Length

18

## Meta Information from PEAKS

### Scan Identifier

F1:3850

### Original Sequence (length=18)

D

P

P

K

T

S

T

S

P

L

V

K

S

F

N

R

N

Q

### Posttranslational Modifications

### Source File

20191211\_F1\_Ag5\_peng0013\_SA\_Flag\_Asp\_N.raw

### Fraction

1

### Scan Feature

F1:1889

### De Novo Score

94

### Confidence score

94

### Mass Charge Ratio

404.019

### Mass

2015.0435

### Charge

5

### Retention Time

21.04

### Predicted Retention Time

-

### Area

190290

### Parts Per Million

7.6

### Fragmentation Mode

ETHCD
