# Supplementary material for: Template-Based Assembly of Proteomic Short Reads For De Novo Antibody Sequencing and Repertoire Profiling
Source: Anal Chem. 2022 Jul 14;94(29):10391–9. doi: 10.1021/acs.analchem.2c01300 (PMC9330293; doi:10.1021/acs.analchem.2c01300)
Supplement: Supplementary file 2 — ac2c01300_si_002.zip [file ac2c01300_si_002.zip › Schulte_2022_ACS-AC_Stitch_SupplementaryData/2022-06-22@17-20-24 anti-FLAG-M2/report-monoclonal/reads/F1_3865.html]

Details F1\_3865

OverviewUndefined

# Read F1:3865

## Sequence

DQEVCKNYAEAK

## Sequence Length

12

## Meta Information from PEAKS

### Scan Identifier

F1:3865

### Original Sequence (length=20)

D

Q

E

V

C

+58.01

K

N

Y

A

E

A

K

### Posttranslational Modifications

Carboxymethyl

### Source File

20191211\_F1\_Ag5\_peng0013\_SA\_Flag\_Asp\_N.raw

### Fraction

1

### Scan Feature

F1:4614

### De Novo Score

99

### Confidence score

99

### Mass Charge Ratio

485.8855

### Mass

1454.6348

### Charge

3

### Retention Time

21.15

### Predicted Retention Time

-

### Area

595040

### Fragmentation Mode

ETHCD

### Also found in scans

F1:3870
