# Supplementary material for: Template-Based Assembly of Proteomic Short Reads For De Novo Antibody Sequencing and Repertoire Profiling
Source: Anal Chem. 2022 Jul 14;94(29):10391–9. doi: 10.1021/acs.analchem.2c01300 (PMC9330293; doi:10.1021/acs.analchem.2c01300)
Supplement: Supplementary file 2 — ac2c01300_si_002.zip [file ac2c01300_si_002.zip › Schulte_2022_ACS-AC_Stitch_SupplementaryData/2022-06-22@17-20-24 anti-FLAG-M2/report-monoclonal/reads/F1_3889.html]

Details F1\_3889

OverviewUndefined

# Read F1:3889

## Sequence

DASTDQDSK

## Sequence Length

9

## Meta Information from PEAKS

### Scan Identifier

F1:3889

### Original Sequence (length=9)

D

A

S

T

D

Q

D

S

K

### Posttranslational Modifications

### Source File

20191211\_F1\_Ag5\_peng0013\_SA\_Flag\_Asp\_N.raw

### Fraction

1

### Scan Feature

F1:4562

### De Novo Score

91

### Confidence score

91

### Mass Charge Ratio

483.7115

### Mass

965.3937

### Charge

2

### Retention Time

21.2

### Predicted Retention Time

-

### Area

651630

### Parts Per Million

15.3

### Fragmentation Mode

ETHCD
