# Supplementary material for: Template-Based Assembly of Proteomic Short Reads For De Novo Antibody Sequencing and Repertoire Profiling
Source: Anal Chem. 2022 Jul 14;94(29):10391–9. doi: 10.1021/acs.analchem.2c01300 (PMC9330293; doi:10.1021/acs.analchem.2c01300)
Supplement: Supplementary file 2 — ac2c01300_si_002.zip [file ac2c01300_si_002.zip › Schulte_2022_ACS-AC_Stitch_SupplementaryData/2022-06-22@17-20-24 anti-FLAG-M2/report-monoclonal/reads/F1_4088.html]

Details F1\_4088

OverviewUndefined

# Read F1:4088

## Sequence

DQASLSCRSSQSLVHRHN

## Sequence Length

18

## Meta Information from PEAKS

### Scan Identifier

F1:4088

### Original Sequence (length=34)

D

Q

A

S

L

S

C

+58.01

R

S

S

Q

S

L

V

H

R

H

+15.99

N

### Posttranslational Modifications

Carboxymethyl; Oxidation (HW)

### Source File

20191211\_F1\_Ag5\_peng0013\_SA\_Flag\_Asp\_N.raw

### Fraction

1

### Scan Feature

F1:11955

### De Novo Score

97

### Confidence score

97

### Mass Charge Ratio

700.3276

### Mass

2097.9609

### Charge

3

### Retention Time

22.42

### Predicted Retention Time

-

### Area

932000

### Parts Per Million

0.1

### Fragmentation Mode

HCD
