# Supplementary material for: Template-Based Assembly of Proteomic Short Reads For De Novo Antibody Sequencing and Repertoire Profiling
Source: Anal Chem. 2022 Jul 14;94(29):10391–9. doi: 10.1021/acs.analchem.2c01300 (PMC9330293; doi:10.1021/acs.analchem.2c01300)
Supplement: Supplementary file 2 — ac2c01300_si_002.zip [file ac2c01300_si_002.zip › Schulte_2022_ACS-AC_Stitch_SupplementaryData/2022-06-22@17-20-24 anti-FLAG-M2/report-monoclonal/reads/F1_4141.html]

Details F1\_4141

OverviewUndefined

# Read F1:4141

## Sequence

DEYERHNSYTCEA

## Sequence Length

13

## Meta Information from PEAKS

### Scan Identifier

F1:4141

### Original Sequence (length=21)

D

E

Y

E

R

H

N

S

Y

T

C

+58.01

E

A

### Posttranslational Modifications

Carboxymethyl

### Source File

20191211\_F1\_Ag5\_peng0013\_SA\_Flag\_Asp\_N.raw

### Fraction

1

### Scan Feature

F1:6631

### De Novo Score

98

### Confidence score

98

### Mass Charge Ratio

558.8832

### Mass

1673.6265

### Charge

3

### Retention Time

22.7

### Predicted Retention Time

-

### Area

183980

### Parts Per Million

0.7

### Fragmentation Mode

ETHCD
