# Supplementary material for: Template-Based Assembly of Proteomic Short Reads For De Novo Antibody Sequencing and Repertoire Profiling
Source: Anal Chem. 2022 Jul 14;94(29):10391–9. doi: 10.1021/acs.analchem.2c01300 (PMC9330293; doi:10.1021/acs.analchem.2c01300)
Supplement: Supplementary file 2 — ac2c01300_si_002.zip [file ac2c01300_si_002.zip › Schulte_2022_ACS-AC_Stitch_SupplementaryData/2022-06-22@17-20-24 anti-FLAG-M2/report-monoclonal/reads/F1_4150.html]

Details F1\_4150

OverviewUndefined

# Read F1:4150

## Sequence

DNQRVLVGG

## Sequence Length

9

## Meta Information from PEAKS

### Scan Identifier

F1:4150

### Original Sequence (length=9)

D

N

Q

R

V

L

V

G

G

### Posttranslational Modifications

### Source File

20191211\_F1\_Ag5\_peng0013\_SA\_Flag\_Asp\_N.raw

### Fraction

1

### Scan Feature

F1:4462

### De Novo Score

98

### Confidence score

98

### Mass Charge Ratio

479.2597

### Mass

956.5039

### Charge

2

### Retention Time

22.79

### Predicted Retention Time

-

### Area

275050

### Parts Per Million

1

### Fragmentation Mode

ETHCD
