# Supplementary material for: Template-Based Assembly of Proteomic Short Reads For De Novo Antibody Sequencing and Repertoire Profiling
Source: Anal Chem. 2022 Jul 14;94(29):10391–9. doi: 10.1021/acs.analchem.2c01300 (PMC9330293; doi:10.1021/acs.analchem.2c01300)
Supplement: Supplementary file 2 — ac2c01300_si_002.zip [file ac2c01300_si_002.zip › Schulte_2022_ACS-AC_Stitch_SupplementaryData/2022-06-22@17-20-24 anti-FLAG-M2/report-monoclonal/reads/F1_4244.html]

Details F1\_4244

OverviewUndefined

# Read F1:4244

## Sequence

DKVRALEEANA

## Sequence Length

11

## Meta Information from PEAKS

### Scan Identifier

F1:4244

### Original Sequence (length=11)

D

K

V

R

A

L

E

E

A

N

A

### Posttranslational Modifications

### Source File

20191211\_F1\_Ag5\_peng0013\_SA\_Flag\_Asp\_N.raw

### Fraction

1

### Scan Feature

F1:1964

### De Novo Score

98

### Confidence score

98

### Mass Charge Ratio

405.8828

### Mass

1214.6255

### Charge

3

### Retention Time

23.35

### Predicted Retention Time

-

### Area

412650

### Parts Per Million

0.9

### Fragmentation Mode

ETHCD
