# Supplementary material for: Template-Based Assembly of Proteomic Short Reads For De Novo Antibody Sequencing and Repertoire Profiling
Source: Anal Chem. 2022 Jul 14;94(29):10391–9. doi: 10.1021/acs.analchem.2c01300 (PMC9330293; doi:10.1021/acs.analchem.2c01300)
Supplement: Supplementary file 2 — ac2c01300_si_002.zip [file ac2c01300_si_002.zip › Schulte_2022_ACS-AC_Stitch_SupplementaryData/2022-06-22@17-20-24 anti-FLAG-M2/report-monoclonal/reads/F1_4266.html]

Details F1\_4266

OverviewUndefined

# Read F1:4266

## Sequence

DKHKTSTSPLVKSFNRNEA

## Sequence Length

19

## Meta Information from PEAKS

### Scan Identifier

F1:4266

### Original Sequence (length=27)

D

K

+58.01

H

K

T

S

T

S

P

L

V

K

S

F

N

R

N

E

A

### Posttranslational Modifications

Carboxymethyl (KW X@N-term)

### Source File

20191211\_F1\_Ag5\_peng0013\_SA\_Flag\_Asp\_N.raw

### Fraction

1

### Scan Feature

F1:3403

### De Novo Score

94

### Confidence score

94

### Mass Charge Ratio

444.2311

### Mass

2216.1184

### Charge

5

### Retention Time

23.37

### Predicted Retention Time

-

### Area

1741500

### Parts Per Million

0.3

### Fragmentation Mode

ETHCD

### Also found in scans

F1:4284
