# Supplementary material for: Template-Based Assembly of Proteomic Short Reads For De Novo Antibody Sequencing and Repertoire Profiling
Source: Anal Chem. 2022 Jul 14;94(29):10391–9. doi: 10.1021/acs.analchem.2c01300 (PMC9330293; doi:10.1021/acs.analchem.2c01300)
Supplement: Supplementary file 2 — ac2c01300_si_002.zip [file ac2c01300_si_002.zip › Schulte_2022_ACS-AC_Stitch_SupplementaryData/2022-06-22@17-20-24 anti-FLAG-M2/report-monoclonal/reads/F1_4366.html]

Details F1\_4366

OverviewUndefined

# Read F1:4366

## Sequence

DGQPAENYKDTQPLM

## Sequence Length

15

## Meta Information from PEAKS

### Scan Identifier

F1:4366

### Original Sequence (length=23)

D

G

Q

P

A

E

N

Y

K

D

T

Q

P

L

M

+15.99

### Posttranslational Modifications

Oxidation (M)

### Source File

20191211\_F1\_Ag5\_peng0013\_SA\_Flag\_Asp\_N.raw

### Fraction

1

### Scan Feature

F1:17160

### De Novo Score

95

### Confidence score

95

### Mass Charge Ratio

861.887

### Mass

1721.7566

### Charge

2

### Retention Time

23.99

### Predicted Retention Time

-

### Area

76262

### Parts Per Million

1.6

### Fragmentation Mode

ETHCD
