# Supplementary material for: Template-Based Assembly of Proteomic Short Reads For De Novo Antibody Sequencing and Repertoire Profiling
Source: Anal Chem. 2022 Jul 14;94(29):10391–9. doi: 10.1021/acs.analchem.2c01300 (PMC9330293; doi:10.1021/acs.analchem.2c01300)
Supplement: Supplementary file 2 — ac2c01300_si_002.zip [file ac2c01300_si_002.zip › Schulte_2022_ACS-AC_Stitch_SupplementaryData/2022-06-22@17-20-24 anti-FLAG-M2/report-monoclonal/reads/F1_4381.html]

Details F1\_4381

OverviewUndefined

# Read F1:4381

## Sequence

DGQPAENYKNTQLW

## Sequence Length

14

## Meta Information from PEAKS

### Scan Identifier

F1:4381

### Original Sequence (length=22)

D

G

Q

P

A

E

N

Y

K

N

T

Q

L

W

+58.01

### Posttranslational Modifications

Carboxymethyl (KW X@N-term)

### Source File

20191211\_F1\_Ag5\_peng0013\_SA\_Flag\_Asp\_N.raw

### Fraction

1

### Scan Feature

F1:7172

### De Novo Score

93

### Confidence score

93

### Mass Charge Ratio

574.5983

### Mass

1720.7693

### Charge

3

### Retention Time

24.29

### Predicted Retention Time

-

### Area

5597000

### Parts Per Million

2.2

### Fragmentation Mode

ETHCD
