# Supplementary material for: Template-Based Assembly of Proteomic Short Reads For De Novo Antibody Sequencing and Repertoire Profiling
Source: Anal Chem. 2022 Jul 14;94(29):10391–9. doi: 10.1021/acs.analchem.2c01300 (PMC9330293; doi:10.1021/acs.analchem.2c01300)
Supplement: Supplementary file 2 — ac2c01300_si_002.zip [file ac2c01300_si_002.zip › Schulte_2022_ACS-AC_Stitch_SupplementaryData/2022-06-22@17-20-24 anti-FLAG-M2/report-monoclonal/reads/F1_4384.html]

Details F1\_4384

OverviewUndefined

# Read F1:4384

## Sequence

DLKVRAL

## Sequence Length

7

## Meta Information from PEAKS

### Scan Identifier

F1:4384

### Original Sequence (length=7)

D

L

K

V

R

A

L

### Posttranslational Modifications

### Source File

20191211\_F1\_Ag5\_peng0013\_SA\_Flag\_Asp\_N.raw

### Fraction

1

### Scan Feature

F1:2033

### De Novo Score

91

### Confidence score

91

### Mass Charge Ratio

407.7613

### Mass

813.5072

### Charge

2

### Retention Time

24.1

### Predicted Retention Time

-

### Area

95907

### Parts Per Million

0.9

### Fragmentation Mode

HCD
