# Supplementary material for: Template-Based Assembly of Proteomic Short Reads For De Novo Antibody Sequencing and Repertoire Profiling
Source: Anal Chem. 2022 Jul 14;94(29):10391–9. doi: 10.1021/acs.analchem.2c01300 (PMC9330293; doi:10.1021/acs.analchem.2c01300)
Supplement: Supplementary file 2 — ac2c01300_si_002.zip [file ac2c01300_si_002.zip › Schulte_2022_ACS-AC_Stitch_SupplementaryData/2022-06-22@17-20-24 anti-FLAG-M2/report-monoclonal/reads/F1_4696.html]

Details F1\_4696

OverviewUndefined

# Read F1:4696

## Sequence

DSAVYYAAR

## Sequence Length

9

## Meta Information from PEAKS

### Scan Identifier

F1:4696

### Original Sequence (length=9)

D

S

A

V

Y

Y

A

A

R

### Posttranslational Modifications

### Source File

20191211\_F1\_Ag5\_peng0013\_SA\_Flag\_Asp\_N.raw

### Fraction

1

### Scan Feature

F1:5187

### De Novo Score

99

### Confidence score

99

### Mass Charge Ratio

508.2462

### Mass

1014.477

### Charge

2

### Retention Time

25.87

### Predicted Retention Time

-

### Area

5698100

### Parts Per Million

0.8

### Fragmentation Mode

HCD
