# Supplementary material for: Template-Based Assembly of Proteomic Short Reads For De Novo Antibody Sequencing and Repertoire Profiling
Source: Anal Chem. 2022 Jul 14;94(29):10391–9. doi: 10.1021/acs.analchem.2c01300 (PMC9330293; doi:10.1021/acs.analchem.2c01300)
Supplement: Supplementary file 2 — ac2c01300_si_002.zip [file ac2c01300_si_002.zip › Schulte_2022_ACS-AC_Stitch_SupplementaryData/2022-06-22@17-20-24 anti-FLAG-M2/report-monoclonal/reads/F1_4724.html]

Details F1\_4724

OverviewUndefined

# Read F1:4724

## Sequence

DPTTVTCNVAHPASSTKV

## Sequence Length

18

## Meta Information from PEAKS

### Scan Identifier

F1:4724

### Original Sequence (length=26)

D

P

T

T

V

T

C

+58.01

N

V

A

H

P

A

S

S

T

K

V

### Posttranslational Modifications

Carboxymethyl

### Source File

20191211\_F1\_Ag5\_peng0013\_SA\_Flag\_Asp\_N.raw

### Fraction

1

### Scan Feature

F1:9425

### De Novo Score

98

### Confidence score

98

### Mass Charge Ratio

629.304

### Mass

1884.8887

### Charge

3

### Retention Time

26.06

### Predicted Retention Time

-

### Area

4598400

### Parts Per Million

0.7

### Fragmentation Mode

ETHCD
