# Supplementary material for: Template-Based Assembly of Proteomic Short Reads For De Novo Antibody Sequencing and Repertoire Profiling
Source: Anal Chem. 2022 Jul 14;94(29):10391–9. doi: 10.1021/acs.analchem.2c01300 (PMC9330293; doi:10.1021/acs.analchem.2c01300)
Supplement: Supplementary file 2 — ac2c01300_si_002.zip [file ac2c01300_si_002.zip › Schulte_2022_ACS-AC_Stitch_SupplementaryData/2022-06-22@17-20-24 anti-FLAG-M2/report-monoclonal/reads/F1_4731.html]

Details F1\_4731

OverviewUndefined

# Read F1:4731

## Sequence

DMEATHKTSTSPLVKSFNRNEC

## Sequence Length

22

## Meta Information from PEAKS

### Scan Identifier

F1:4731

### Original Sequence (length=38)

D

M

+15.99

E

A

T

H

K

T

S

T

S

P

L

V

K

S

F

N

R

N

E

C

+58.01

### Posttranslational Modifications

Oxidation (M); Carboxymethyl

### Source File

20191211\_F1\_Ag5\_peng0013\_SA\_Flag\_Asp\_N.raw

### Fraction

1

### Scan Feature

F1:10097

### De Novo Score

96

### Confidence score

96

### Mass Charge Ratio

643.0469

### Mass

2568.1584

### Charge

4

### Retention Time

26.11

### Predicted Retention Time

-

### Area

1285700

### Parts Per Million

0

### Fragmentation Mode

ETHCD
