# Supplementary material for: Template-Based Assembly of Proteomic Short Reads For De Novo Antibody Sequencing and Repertoire Profiling
Source: Anal Chem. 2022 Jul 14;94(29):10391–9. doi: 10.1021/acs.analchem.2c01300 (PMC9330293; doi:10.1021/acs.analchem.2c01300)
Supplement: Supplementary file 2 — ac2c01300_si_002.zip [file ac2c01300_si_002.zip › Schulte_2022_ACS-AC_Stitch_SupplementaryData/2022-06-22@17-20-24 anti-FLAG-M2/report-monoclonal/reads/F1_4777.html]

Details F1\_4777

OverviewUndefined

# Read F1:4777

## Sequence

DGYMDSLHTSRNTYTA

## Sequence Length

16

## Meta Information from PEAKS

### Scan Identifier

F1:4777

### Original Sequence (length=24)

D

G

Y

M

+15.99

D

S

L

H

T

S

R

N

T

Y

T

A

### Posttranslational Modifications

Oxidation (M)

### Source File

20191211\_F1\_Ag5\_peng0013\_SA\_Flag\_Asp\_N.raw

### Fraction

1

### Scan Feature

F1:8721

### De Novo Score

99

### Confidence score

99

### Mass Charge Ratio

616.6006

### Mass

1846.7793

### Charge

3

### Retention Time

26.37

### Predicted Retention Time

-

### Area

3152500

### Parts Per Million

0.3

### Fragmentation Mode

ETHCD

### Also found in scans

F1:4667
