# Supplementary material for: Template-Based Assembly of Proteomic Short Reads For De Novo Antibody Sequencing and Repertoire Profiling
Source: Anal Chem. 2022 Jul 14;94(29):10391–9. doi: 10.1021/acs.analchem.2c01300 (PMC9330293; doi:10.1021/acs.analchem.2c01300)
Supplement: Supplementary file 2 — ac2c01300_si_002.zip [file ac2c01300_si_002.zip › Schulte_2022_ACS-AC_Stitch_SupplementaryData/2022-06-22@17-20-24 anti-FLAG-M2/report-monoclonal/reads/F1_4926.html]

Details F1\_4926

OverviewUndefined

# Read F1:4926

## Sequence

DLGGVFAQKLDGSERQ

## Sequence Length

16

## Meta Information from PEAKS

### Scan Identifier

F1:4926

### Original Sequence (length=16)

D

L

G

G

V

F

A

Q

K

L

D

G

S

E

R

Q

### Posttranslational Modifications

### Source File

20191211\_F1\_Ag5\_peng0013\_SA\_Flag\_Asp\_N.raw

### Fraction

1

### Scan Feature

F1:2937

### De Novo Score

92

### Confidence score

92

### Mass Charge Ratio

430.7222

### Mass

1718.8586

### Charge

4

### Retention Time

27.26

### Predicted Retention Time

-

### Area

4128200

### Parts Per Million

0.7

### Fragmentation Mode

HCD
