# Supplementary material for: Template-Based Assembly of Proteomic Short Reads For De Novo Antibody Sequencing and Repertoire Profiling
Source: Anal Chem. 2022 Jul 14;94(29):10391–9. doi: 10.1021/acs.analchem.2c01300 (PMC9330293; doi:10.1021/acs.analchem.2c01300)
Supplement: Supplementary file 2 — ac2c01300_si_002.zip [file ac2c01300_si_002.zip › Schulte_2022_ACS-AC_Stitch_SupplementaryData/2022-06-22@17-20-24 anti-FLAG-M2/report-monoclonal/reads/F1_4937.html]

Details F1\_4937

OverviewUndefined

# Read F1:4937

## Sequence

DLNVKMAKLDGSERQ

## Sequence Length

15

## Meta Information from PEAKS

### Scan Identifier

F1:4937

### Original Sequence (length=23)

D

L

N

V

K

M

+15.99

A

K

L

D

G

S

E

R

Q

### Posttranslational Modifications

Oxidation (M)

### Source File

20191211\_F1\_Ag5\_peng0013\_SA\_Flag\_Asp\_N.raw

### Fraction

1

### Scan Feature

F1:7147

### De Novo Score

94

### Confidence score

94

### Mass Charge Ratio

573.9604

### Mass

1718.8621

### Charge

3

### Retention Time

27.26

### Predicted Retention Time

-

### Area

10523000

### Fragmentation Mode

HCD

### Also found in scans

F1:5227
