# Supplementary material for: Template-Based Assembly of Proteomic Short Reads For De Novo Antibody Sequencing and Repertoire Profiling
Source: Anal Chem. 2022 Jul 14;94(29):10391–9. doi: 10.1021/acs.analchem.2c01300 (PMC9330293; doi:10.1021/acs.analchem.2c01300)
Supplement: Supplementary file 2 — ac2c01300_si_002.zip [file ac2c01300_si_002.zip › Schulte_2022_ACS-AC_Stitch_SupplementaryData/2022-06-22@17-20-24 anti-FLAG-M2/report-monoclonal/reads/F1_4967.html]

Details F1\_4967

OverviewUndefined

# Read F1:4967

## Sequence

DDVHKTSTSPLVKSFNRNEC

## Sequence Length

20

## Meta Information from PEAKS

### Scan Identifier

F1:4967

### Original Sequence (length=28)

D

D

V

H

K

T

S

T

S

P

L

V

K

S

F

N

R

N

E

C

+58.01

### Posttranslational Modifications

Carboxymethyl

### Source File

20191211\_F1\_Ag5\_peng0013\_SA\_Flag\_Asp\_N.raw

### Fraction

1

### Scan Feature

F1:7491

### De Novo Score

93

### Confidence score

93

### Mass Charge Ratio

584.5297

### Mass

2334.0908

### Charge

4

### Retention Time

27.57

### Predicted Retention Time

-

### Area

9828700

### Fragmentation Mode

ETHCD
