# Supplementary material for: Template-Based Assembly of Proteomic Short Reads For De Novo Antibody Sequencing and Repertoire Profiling
Source: Anal Chem. 2022 Jul 14;94(29):10391–9. doi: 10.1021/acs.analchem.2c01300 (PMC9330293; doi:10.1021/acs.analchem.2c01300)
Supplement: Supplementary file 2 — ac2c01300_si_002.zip [file ac2c01300_si_002.zip › Schulte_2022_ACS-AC_Stitch_SupplementaryData/2022-06-22@17-20-24 anti-FLAG-M2/report-monoclonal/reads/F1_5025.html]

Details F1\_5025

OverviewUndefined

# Read F1:5025

## Sequence

DMRHKTSTSPLVKSFNRNEC

## Sequence Length

20

## Meta Information from PEAKS

### Scan Identifier

F1:5025

### Original Sequence (length=44)

D

+58.01

M

+15.99

R

H

K

T

S

T

S

P

L

V

K

S

F

N

R

N

E

C

+58.01

### Posttranslational Modifications

Carboxymethyl (KW X@N-term); Oxidation (M); Carboxymethyl

### Source File

20191211\_F1\_Ag5\_peng0013\_SA\_Flag\_Asp\_N.raw

### Fraction

1

### Scan Feature

F1:8937

### De Novo Score

91

### Confidence score

91

### Mass Charge Ratio

621.293

### Mass

2481.1375

### Charge

4

### Retention Time

27.72

### Predicted Retention Time

-

### Area

9472700

### Parts Per Million

2.1

### Fragmentation Mode

ETHCD
