# Supplementary material for: Template-Based Assembly of Proteomic Short Reads For De Novo Antibody Sequencing and Repertoire Profiling
Source: Anal Chem. 2022 Jul 14;94(29):10391–9. doi: 10.1021/acs.analchem.2c01300 (PMC9330293; doi:10.1021/acs.analchem.2c01300)
Supplement: Supplementary file 2 — ac2c01300_si_002.zip [file ac2c01300_si_002.zip › Schulte_2022_ACS-AC_Stitch_SupplementaryData/2022-06-22@17-20-24 anti-FLAG-M2/report-monoclonal/reads/F1_5067.html]

Details F1\_5067

OverviewUndefined

# Read F1:5067

## Sequence

DEYERHNSYTCEATHKTSTKLPKSFHRG

## Sequence Length

28

## Meta Information from PEAKS

### Scan Identifier

F1:5067

### Original Sequence (length=52)

D

E

Y

E

R

H

N

S

Y

T

C

+58.01

E

A

T

H

K

T

S

T

K

+58.01

L

P

K

S

F

H

+15.99

R

G

### Posttranslational Modifications

Carboxymethyl; Carboxymethyl (KW X@N-term); Oxidation (HW)

### Source File

20191211\_F1\_Ag5\_peng0013\_SA\_Flag\_Asp\_N.raw

### Fraction

1

### Scan Feature

F1:17224

### De Novo Score

90

### Confidence score

90

### Mass Charge Ratio

864.3981

### Mass

3453.5642

### Charge

4

### Retention Time

27.93

### Predicted Retention Time

-

### Area

2346900

### Fragmentation Mode

ETHCD
