# Supplementary material for: Template-Based Assembly of Proteomic Short Reads For De Novo Antibody Sequencing and Repertoire Profiling
Source: Anal Chem. 2022 Jul 14;94(29):10391–9. doi: 10.1021/acs.analchem.2c01300 (PMC9330293; doi:10.1021/acs.analchem.2c01300)
Supplement: Supplementary file 2 — ac2c01300_si_002.zip [file ac2c01300_si_002.zip › Schulte_2022_ACS-AC_Stitch_SupplementaryData/2022-06-22@17-20-24 anti-FLAG-M2/report-monoclonal/reads/F1_5357.html]

Details F1\_5357

OverviewUndefined

# Read F1:5357

## Sequence

DGVLNSAMTDQDSK

## Sequence Length

14

## Meta Information from PEAKS

### Scan Identifier

F1:5357

### Original Sequence (length=22)

D

G

V

L

N

S

A

M

+15.99

T

D

Q

D

S

K

### Posttranslational Modifications

Oxidation (M)

### Source File

20191211\_F1\_Ag5\_peng0013\_SA\_Flag\_Asp\_N.raw

### Fraction

1

### Scan Feature

F1:13660

### De Novo Score

94

### Confidence score

94

### Mass Charge Ratio

748.8287

### Mass

1495.646

### Charge

2

### Retention Time

29.51

### Predicted Retention Time

-

### Area

3898800

### Fragmentation Mode

HCD
