# Supplementary material for: Template-Based Assembly of Proteomic Short Reads For De Novo Antibody Sequencing and Repertoire Profiling
Source: Anal Chem. 2022 Jul 14;94(29):10391–9. doi: 10.1021/acs.analchem.2c01300 (PMC9330293; doi:10.1021/acs.analchem.2c01300)
Supplement: Supplementary file 2 — ac2c01300_si_002.zip [file ac2c01300_si_002.zip › Schulte_2022_ACS-AC_Stitch_SupplementaryData/2022-06-22@17-20-24 anti-FLAG-M2/report-monoclonal/reads/F1_5389.html]

Details F1\_5389

OverviewUndefined

# Read F1:5389

## Sequence

DQDSKDSTYSMSSTLTLTK

## Sequence Length

19

## Meta Information from PEAKS

### Scan Identifier

F1:5389

### Original Sequence (length=27)

D

Q

D

S

K

D

S

T

Y

S

M

+15.99

S

S

T

L

T

L

T

K

### Posttranslational Modifications

Oxidation (M)

### Source File

20191211\_F1\_Ag5\_peng0013\_SA\_Flag\_Asp\_N.raw

### Fraction

1

### Scan Feature

F1:12313

### De Novo Score

99

### Confidence score

99

### Mass Charge Ratio

708.6602

### Mass

2122.9575

### Charge

3

### Retention Time

29.56

### Predicted Retention Time

-

### Area

88205000

### Parts Per Million

0.5

### Fragmentation Mode

ETHCD

### Also found in scans

F1:5341 F1:5584
