# Supplementary material for: Template-Based Assembly of Proteomic Short Reads For De Novo Antibody Sequencing and Repertoire Profiling
Source: Anal Chem. 2022 Jul 14;94(29):10391–9. doi: 10.1021/acs.analchem.2c01300 (PMC9330293; doi:10.1021/acs.analchem.2c01300)
Supplement: Supplementary file 2 — ac2c01300_si_002.zip [file ac2c01300_si_002.zip › Schulte_2022_ACS-AC_Stitch_SupplementaryData/2022-06-22@17-20-24 anti-FLAG-M2/report-monoclonal/reads/F1_5433.html]

Details F1\_5433

OverviewUndefined

# Read F1:5433

## Sequence

DSTYSQETLTLTK

## Sequence Length

13

## Meta Information from PEAKS

### Scan Identifier

F1:5433

### Original Sequence (length=13)

D

S

T

Y

S

Q

E

T

L

T

L

T

K

### Posttranslational Modifications

### Source File

20191211\_F1\_Ag5\_peng0013\_SA\_Flag\_Asp\_N.raw

### Fraction

1

### Scan Feature

F1:4870

### De Novo Score

97

### Confidence score

97

### Mass Charge Ratio

496.2474

### Mass

1485.72

### Charge

3

### Retention Time

29.66

### Predicted Retention Time

-

### Area

137130000

### Parts Per Million

0.3

### Fragmentation Mode

ETHCD

### Also found in scans

F4:5399 F4:5458 F1:5505
