# Supplementary material for: Template-Based Assembly of Proteomic Short Reads For De Novo Antibody Sequencing and Repertoire Profiling
Source: Anal Chem. 2022 Jul 14;94(29):10391–9. doi: 10.1021/acs.analchem.2c01300 (PMC9330293; doi:10.1021/acs.analchem.2c01300)
Supplement: Supplementary file 2 — ac2c01300_si_002.zip [file ac2c01300_si_002.zip › Schulte_2022_ACS-AC_Stitch_SupplementaryData/2022-06-22@17-20-24 anti-FLAG-M2/report-monoclonal/reads/F1_5437.html]

Details F1\_5437

OverviewUndefined

# Read F1:5437

## Sequence

DLNVKWKLDGSERQ

## Sequence Length

14

## Meta Information from PEAKS

### Scan Identifier

F1:5437

### Original Sequence (length=22)

D

L

N

V

K

W

+15.99

K

L

D

G

S

E

R

Q

### Posttranslational Modifications

Oxidation (HW)

### Source File

20191211\_F1\_Ag5\_peng0013\_SA\_Flag\_Asp\_N.raw

### Fraction

1

### Scan Feature

F1:2784

### De Novo Score

99

### Confidence score

99

### Mass Charge Ratio

426.724

### Mass

1702.8638

### Charge

4

### Retention Time

30.03

### Predicted Retention Time

-

### Area

1515500

### Parts Per Million

1.9

### Fragmentation Mode

HCD

### Also found in scans

F1:6333 F1:4439 F1:6378 F1:4451 F1:6551 F1:5567 F1:6167 F1:6209 F1:6992
