# Supplementary material for: Template-Based Assembly of Proteomic Short Reads For De Novo Antibody Sequencing and Repertoire Profiling
Source: Anal Chem. 2022 Jul 14;94(29):10391–9. doi: 10.1021/acs.analchem.2c01300 (PMC9330293; doi:10.1021/acs.analchem.2c01300)
Supplement: Supplementary file 2 — ac2c01300_si_002.zip [file ac2c01300_si_002.zip › Schulte_2022_ACS-AC_Stitch_SupplementaryData/2022-06-22@17-20-24 anti-FLAG-M2/report-monoclonal/reads/F1_5589.html]

Details F1\_5589

OverviewUndefined

# Read F1:5589

## Sequence

DSTYEMSSTLTLTK

## Sequence Length

14

## Meta Information from PEAKS

### Scan Identifier

F1:5589

### Original Sequence (length=22)

D

S

T

Y

E

M

+15.99

S

S

T

L

T

L

T

K

### Posttranslational Modifications

Oxidation (M)

### Source File

20191211\_F1\_Ag5\_peng0013\_SA\_Flag\_Asp\_N.raw

### Fraction

1

### Scan Feature

-

### De Novo Score

93

### Confidence score

93

### Mass Charge Ratio

531.5836

### Mass

1591.7288

### Charge

3

### Retention Time

30.93

### Predicted Retention Time

-

### Area

0

### Parts Per Million

0.1

### Fragmentation Mode

ETHCD

### Also found in scans

F1:5533 F1:5737
