# Supplementary material for: Template-Based Assembly of Proteomic Short Reads For De Novo Antibody Sequencing and Repertoire Profiling
Source: Anal Chem. 2022 Jul 14;94(29):10391–9. doi: 10.1021/acs.analchem.2c01300 (PMC9330293; doi:10.1021/acs.analchem.2c01300)
Supplement: Supplementary file 2 — ac2c01300_si_002.zip [file ac2c01300_si_002.zip › Schulte_2022_ACS-AC_Stitch_SupplementaryData/2022-06-22@17-20-24 anti-FLAG-M2/report-monoclonal/reads/F1_5776.html]

Details F1\_5776

OverviewUndefined

# Read F1:5776

## Sequence

DSYTCEATHKTSTSPLVKSLMMTAPC

## Sequence Length

26

## Meta Information from PEAKS

### Scan Identifier

F1:5776

### Original Sequence (length=50)

D

S

Y

T

C

+58.01

E

A

T

H

K

T

S

T

S

P

L

V

K

S

L

M

M

+15.99

T

A

P

C

+58.01

### Posttranslational Modifications

Carboxymethyl; Oxidation (M)

### Source File

20191211\_F1\_Ag5\_peng0013\_SA\_Flag\_Asp\_N.raw

### Fraction

1

### Scan Feature

F1:7613

### De Novo Score

90

### Confidence score

90

### Mass Charge Ratio

587.6635

### Mass

2933.2803

### Charge

5

### Retention Time

32.14

### Predicted Retention Time

-

### Area

33847000

### Parts Per Million

0.2

### Fragmentation Mode

HCD
