# Supplementary material for: Template-Based Assembly of Proteomic Short Reads For De Novo Antibody Sequencing and Repertoire Profiling
Source: Anal Chem. 2022 Jul 14;94(29):10391–9. doi: 10.1021/acs.analchem.2c01300 (PMC9330293; doi:10.1021/acs.analchem.2c01300)
Supplement: Supplementary file 2 — ac2c01300_si_002.zip [file ac2c01300_si_002.zip › Schulte_2022_ACS-AC_Stitch_SupplementaryData/2022-06-22@17-20-24 anti-FLAG-M2/report-monoclonal/reads/F1_5802.html]

Details F1\_5802

OverviewUndefined

# Read F1:5802

## Sequence

DSYTCEATHKTSTSPLVKSFNNREC

## Sequence Length

25

## Meta Information from PEAKS

### Scan Identifier

F1:5802

### Original Sequence (length=41)

D

S

Y

T

C

+58.01

E

A

T

H

K

T

S

T

S

P

L

V

K

S

F

N

N

R

E

C

+58.01

### Posttranslational Modifications

Carboxymethyl

### Source File

20191211\_F1\_Ag5\_peng0013\_SA\_Flag\_Asp\_N.raw

### Fraction

1

### Scan Feature

F1:20957

### De Novo Score

93

### Confidence score

93

### Mass Charge Ratio

978.7661

### Mass

2933.2808

### Charge

3

### Retention Time

32.13

### Predicted Retention Time

-

### Area

40050000

### Fragmentation Mode

HCD
