# Supplementary material for: Template-Based Assembly of Proteomic Short Reads For De Novo Antibody Sequencing and Repertoire Profiling
Source: Anal Chem. 2022 Jul 14;94(29):10391–9. doi: 10.1021/acs.analchem.2c01300 (PMC9330293; doi:10.1021/acs.analchem.2c01300)
Supplement: Supplementary file 2 — ac2c01300_si_002.zip [file ac2c01300_si_002.zip › Schulte_2022_ACS-AC_Stitch_SupplementaryData/2022-06-22@17-20-24 anti-FLAG-M2/report-monoclonal/reads/F1_6082.html]

Details F1\_6082

OverviewUndefined

# Read F1:6082

## Sequence

DLNVKWKLDGSERQN

## Sequence Length

15

## Meta Information from PEAKS

### Scan Identifier

F1:6082

### Original Sequence (length=15)

D

L

N

V

K

W

K

L

D

G

S

E

R

Q

N

### Posttranslational Modifications

### Source File

20191211\_F1\_Ag5\_peng0013\_SA\_Flag\_Asp\_N.raw

### Fraction

1

### Scan Feature

F1:8122

### De Novo Score

98

### Confidence score

98

### Mass Charge Ratio

601.3112

### Mass

1800.9119

### Charge

3

### Retention Time

33.62

### Predicted Retention Time

-

### Area

9121900

### Fragmentation Mode

HCD

### Also found in scans

F1:6073
