# Supplementary material for: Template-Based Assembly of Proteomic Short Reads For De Novo Antibody Sequencing and Repertoire Profiling
Source: Anal Chem. 2022 Jul 14;94(29):10391–9. doi: 10.1021/acs.analchem.2c01300 (PMC9330293; doi:10.1021/acs.analchem.2c01300)
Supplement: Supplementary file 2 — ac2c01300_si_002.zip [file ac2c01300_si_002.zip › Schulte_2022_ACS-AC_Stitch_SupplementaryData/2022-06-22@17-20-24 anti-FLAG-M2/report-monoclonal/reads/F1_6145.html]

Details F1\_6145

OverviewUndefined

# Read F1:6145

## Sequence

DLNVKWKLDGSWVQ

## Sequence Length

14

## Meta Information from PEAKS

### Scan Identifier

F1:6145

### Original Sequence (length=14)

D

L

N

V

K

W

K

L

D

G

S

W

V

Q

### Posttranslational Modifications

### Source File

20191211\_F1\_Ag5\_peng0013\_SA\_Flag\_Asp\_N.raw

### Fraction

1

### Scan Feature

F1:2619

### De Novo Score

97

### Confidence score

97

### Mass Charge Ratio

422.7244

### Mass

1686.8728

### Charge

4

### Retention Time

34.09

### Predicted Retention Time

-

### Area

157500000

### Fragmentation Mode

HCD
