# Supplementary material for: Template-Based Assembly of Proteomic Short Reads For De Novo Antibody Sequencing and Repertoire Profiling
Source: Anal Chem. 2022 Jul 14;94(29):10391–9. doi: 10.1021/acs.analchem.2c01300 (PMC9330293; doi:10.1021/acs.analchem.2c01300)
Supplement: Supplementary file 2 — ac2c01300_si_002.zip [file ac2c01300_si_002.zip › Schulte_2022_ACS-AC_Stitch_SupplementaryData/2022-06-22@17-20-24 anti-FLAG-M2/report-monoclonal/reads/F1_6147_002.html]

Details F1\_6147\_002

OverviewUndefined

# Read F1:6147\_002

## Sequence

DSEYSMFELTLTK

## Sequence Length

13

## Meta Information from PEAKS

### Scan Identifier

F1:6147

### Original Sequence (length=21)

D

S

E

Y

S

M

+15.99

F

E

L

T

L

T

K

### Posttranslational Modifications

Oxidation (M)

### Source File

20191211\_F1\_Ag5\_peng0013\_SA\_Flag\_Asp\_N.raw

### Fraction

1

### Scan Feature

-

### De Novo Score

96

### Confidence score

96

### Mass Charge Ratio

790.3657

### Mass

1578.7124

### Charge

2

### Retention Time

34.07

### Predicted Retention Time

-

### Area

0

### Parts Per Million

2.8

### Fragmentation Mode

HCD
