# Supplementary material for: Template-Based Assembly of Proteomic Short Reads For De Novo Antibody Sequencing and Repertoire Profiling
Source: Anal Chem. 2022 Jul 14;94(29):10391–9. doi: 10.1021/acs.analchem.2c01300 (PMC9330293; doi:10.1021/acs.analchem.2c01300)
Supplement: Supplementary file 2 — ac2c01300_si_002.zip [file ac2c01300_si_002.zip › Schulte_2022_ACS-AC_Stitch_SupplementaryData/2022-06-22@17-20-24 anti-FLAG-M2/report-monoclonal/reads/F1_6313.html]

Details F1\_6313

OverviewUndefined

# Read F1:6313

## Sequence

DKVSHDCMLT

## Sequence Length

10

## Meta Information from PEAKS

### Scan Identifier

F1:6313

### Original Sequence (length=26)

D

K

V

S

H

D

C

+58.01

M

+15.99

L

T

### Posttranslational Modifications

Carboxymethyl; Oxidation (M)

### Source File

20191211\_F1\_Ag5\_peng0013\_SA\_Flag\_Asp\_N.raw

### Fraction

1

### Scan Feature

F1:2042

### De Novo Score

93

### Confidence score

93

### Mass Charge Ratio

408.1721

### Mass

1221.5005

### Charge

3

### Retention Time

34.97

### Predicted Retention Time

-

### Area

2077400

### Fragmentation Mode

ETHCD
