# Supplementary material for: Template-Based Assembly of Proteomic Short Reads For De Novo Antibody Sequencing and Repertoire Profiling
Source: Anal Chem. 2022 Jul 14;94(29):10391–9. doi: 10.1021/acs.analchem.2c01300 (PMC9330293; doi:10.1021/acs.analchem.2c01300)
Supplement: Supplementary file 2 — ac2c01300_si_002.zip [file ac2c01300_si_002.zip › Schulte_2022_ACS-AC_Stitch_SupplementaryData/2022-06-22@17-20-24 anti-FLAG-M2/report-monoclonal/reads/F1_6473.html]

Details F1\_6473

OverviewUndefined

# Read F1:6473

## Sequence

DPSSSTAYMEL

## Sequence Length

11

## Meta Information from PEAKS

### Scan Identifier

F1:6473

### Original Sequence (length=19)

D

P

S

S

S

T

A

Y

M

+15.99

E

L

### Posttranslational Modifications

Oxidation (M)

### Source File

20191211\_F1\_Ag5\_peng0013\_SA\_Flag\_Asp\_N.raw

### Fraction

1

### Scan Feature

F1:8358

### De Novo Score

96

### Confidence score

96

### Mass Charge Ratio

608.7559

### Mass

1215.4966

### Charge

2

### Retention Time

35.97

### Predicted Retention Time

-

### Area

6159500

### Parts Per Million

0.5

### Fragmentation Mode

HCD
