# Supplementary material for: Template-Based Assembly of Proteomic Short Reads For De Novo Antibody Sequencing and Repertoire Profiling
Source: Anal Chem. 2022 Jul 14;94(29):10391–9. doi: 10.1021/acs.analchem.2c01300 (PMC9330293; doi:10.1021/acs.analchem.2c01300)
Supplement: Supplementary file 2 — ac2c01300_si_002.zip [file ac2c01300_si_002.zip › Schulte_2022_ACS-AC_Stitch_SupplementaryData/2022-06-22@17-20-24 anti-FLAG-M2/report-monoclonal/reads/F1_6689.html]

Details F1\_6689

OverviewUndefined

# Read F1:6689

## Sequence

DLNVKMAKLDGSERQNGVLNSWT

## Sequence Length

23

## Meta Information from PEAKS

### Scan Identifier

F1:6689

### Original Sequence (length=31)

D

L

N

V

K

M

A

K

L

D

G

S

E

R

Q

N

G

V

L

N

S

W

+15.99

T

### Posttranslational Modifications

Oxidation (HW)

### Source File

20191211\_F1\_Ag5\_peng0013\_SA\_Flag\_Asp\_N.raw

### Fraction

1

### Scan Feature

F1:10321

### De Novo Score

91

### Confidence score

91

### Mass Charge Ratio

648.5763

### Mass

2590.2808

### Charge

4

### Retention Time

37.06

### Predicted Retention Time

-

### Area

1039400

### Fragmentation Mode

ETHCD
